# Fundamental solutions for semidiscrete evolution equations via Banach algebras

**DOI:** 10.1186/s13662-020-03206-7

**Published:** 2021-01-07

**Authors:** Jorge González-Camus, Carlos Lizama, Pedro J. Miana

**Affiliations:** 1 Departamento de Matemáticas y Ciencias de la Computación, Facultad de Ciencias, Universidad de Santiago de Chile, Las Sophoras 173, Estación Central, Santiago, Chile; 2 Departamento de Matemáticas, Instituto Universitario de Matemáticas y Aplicaciones, Universidad de Zaragoza, 50009 Zaragoza, Spain

**Keywords:** 35R11, 35A08, 39A12, Caputo fractional derivative, Discrete fractional Laplacian, Discrete fractional operators, Fundamental solutions, Wright and Mittag-Leffler functions

## Abstract

We give representations for solutions of time-fractional differential equations that involve operators on Lebesgue spaces of sequences defined by discrete convolutions involving kernels through the discrete Fourier transform. We consider finite difference operators of first and second orders, which are generators of uniformly continuous semigroups and cosine functions. We present the linear and algebraic structures (in particular, factorization properties) and their norms and spectra in the Lebesgue space of summable sequences. We identify fractional powers of these generators and apply to them the subordination principle. We also give some applications and consequences of our results.

## Introduction

In this work, we study the following semidiscrete Cauchy problem:

1.1 $$ \textstyle\begin{cases} \partial _{t} u(n,t)=Bu(n,t)+g(n,t), &n\in \mathbb{Z}, t>0, \\ u(n,0)=\varphi (n),&n\in \mathbb{Z}, \end{cases} $$

where *B* is the convolution operator in the discrete variable, that is,

1.2 $$ Bu(n,t)= \sum_{j\in \mathbb{Z}} b(n-j)u(j,t) $$

with *b* belonging to the Banach algebra $\ell ^{1}(\mathbb{Z})$. A typical example is the one-dimensional discrete Laplacian $\Delta _{d}$, which can be obtained by taking $b= \delta _{-1} - 2\delta _{0} + \delta _{1}$, where $\delta _{i}(j)$ denotes the Kronecker delta (or discrete Dirac measure). In such a case, equation (1.1) corresponds to the nonhomogeneous semidiscrete diffusion equation (also known as the semidiscrete heat equation or the lattice diffusion equation).

The analytical study of such equations has received an increasing interest in the last decade, mainly due to many their applications in diverse areas of knowledge. For instance, in probability theory, the value $u(n,t)$ in (1.1) with $B=\Delta _{d}$ describes the probability that a continuous-time symmetric random walk on $\mathbb{Z}$ visits a point *n* at time *t*; see [25, Sect. 4]. In chemistry, (1.1) describes the flow of a chemical in an infinite system of tanks arranged in a row, where each two neighbors are connected by pipes [42, Sect. 3], and in transport theory, (1.1) describes the dynamics of an infinite chain of cars, each being coupled to its two neighbors. The value $u(n; t)$ is the displacement of car *n* at time *t* from its equilibrium position; see [24, Example 1]. From an analytical point of view, quite recently, Slavik [43] studied the asymptotic behavior of solutions of (1.1) when $B=\Delta _{d}$, showing that a bounded solution approaches the average of the initial values if the average exists. Note that choosing $b= \delta _{-1}-\delta _{0}$ in (1.2), we obtain the forward difference operator $B=\Delta $, and hence (1.2) corresponds to the semidiscrete transport equation, studied recently by Abadias et al. [1].

It is interesting that in [22] and [37] the authors studied the fundamental solutions of (1.1) and the second-order semidiscrete equation

1.3 $$ \textstyle\begin{cases} \partial _{tt} u(n,t)=Bu(n,t)+g(n,t), &n\in \mathbb{Z}, t>0, \\ u(n,0)=\varphi (n), \qquad u_{t}(n,0)= \phi (n),&n\in \mathbb{Z}, \end{cases} $$

when $B=-(-\Delta _{d})^{\alpha }$ is the discrete fractional Laplacian. Particularly, in [37] the authors combined operator theory techniques with the properties of the Bessel functions to develop a theory of analytic semigroups and cosine operators generated by $\Delta _{d}$ and $-(-\Delta _{d})^{\alpha }$. Also note that the fractional forward difference operator $B=-(-\Delta )^{\alpha }$ was studied in [1], where the maximum and comparison principles in the context of harmonic analysis are proved.

However, to our knowledge, to date, there is no attempt to investigate the fundamental solutions of the general equation (1.1) in a unified way. Our goal in this paper is to propose a solution to this problem.

Our key observation concerning this issue is that the discrete fractional Laplacian can be obtained from (1.2) by allowing the fractional powers of *b* to be an element of the Banach algebra $\ell ^{1}(\mathbb{Z})$. This original approach, which we provide in this paper, allows us to obtain new insights by introducing a completely new method to analyze both qualitative behavior and fundamental solutions of (1.1) in a unified way.

More generally, to provide simultaneously in our analysis the subdiffusive and superdifussive cases associated with equations (1.1) and (1.3), in this paper, we include a representation of the fundamental solutions for the following semidiscrete equations:

1.4 $$ \textstyle\begin{cases} \mathbb{D}_{t}^{\beta }u(n,t)=Bu(n,t)+g(n,t), &n\in \mathbb{Z}, t>0, \\ u(n,0)=\varphi (n),&n\in \mathbb{Z}, \end{cases} $$

in case $0<\beta \leq 1 $ and

1.5 $$ \textstyle\begin{cases} \mathbb{D}_{t}^{\beta }u(n,t)=Bu(n,t)+g(n,t), &n\in \mathbb{Z}, t>0, \\ u(n,0)=\varphi (n),\qquad u_{t}(n,0)= \phi (n),&n\in \mathbb{Z}, \end{cases} $$

in case $1<\beta \leq 2$. In both cases, *B* is the convolution operator $Bf(n):=(b \ast f)(n)$ on $\ell ^{p}(\mathbb{Z})$, $p\in [1,\infty ]$, $b \in \ell ^{1}(\mathbb{Z})$, and $\beta \in (0,2]$. The symbol $\mathbb{D}_{t}^{\beta }$ denotes the Caputo fractional derivative of order $\beta >0$.

We observe that although the present study considers only the cases $0 <\beta \leq 2 $ because they are the most common in applications (that is, subdiffusion and superdiffusion) together with the fractional Caputo derivative, our method is general enough to consider a larger order *β* and other nonlocal operators in time. For example, in Sect. 6.4, we consider the new Caputo–Fabrizio fractional derivative of order $\alpha \in (0,1)$ and give a representation of the solutions for the corresponding equation (1.4).

This paper is organized as follows. In Sect. 2, we consider the Banach algebra framework to state our main results, which we will present in the forthcoming sections. In particular, we introduce generalized Mittag-Leffer functions on the Banach algebra $\ell ^{1}(\mathbb{Z})$ and collect some basic properties. Our main result is Theorem 2.6 concerning the invariance for convolution operators defined on $\ell ^{p}(\mathbb{Z})$ for $1\leq p \leq \infty $. Section 3 is devoted to four finite difference operators: backward and forward difference operators, the one-dimensional discrete Laplacian, and an operator that originates in connection with crystal lattices [16]. Then we explicitly describe their associated groups and cosine operators by means of Bessel functions and highlight their main spectral properties. Section 4 begins with three concrete examples of application of the results in the previous section: the discrete Nagumo equation, transport equations, and a new interesting second-order discrete equation, which we call the De Juhasz equation, appearing in the seminal Bateman’s paper [16] in connection with surges in springs and connected systems of springs. Then we state the general fundamental solutions for (1.4)–(1.5), first, in the setting of Banach algebras (Theorem 5.1) and then for convolution operators (Corollary 5.5). In Sect. 7, we give explicit representations of generalized Mittag-Leffer functions in each case of the fractional powers of the four finite difference operators considered previously (Theorem 7.1). This result, combined with the general fundamental solutions considered in Theorem 5.1 and Corollary 5.5, gives not only explicit representations of each of the four difference operators considered in this paper – which can be considered as examples – but also an efficient method to obtain representations of solutions in many other cases. Besides, as a byproduct of our treatment, we obtain new Weiestrass formulae, which highlight the role of Bessel functions for finite difference operators, and a subordination principle, which connects the Wright and Bessel functions. For convenience of the reader, we finish this research with an appendix on useful properties of some special functions needed in this paper.

*Notation.* $\mathbb{T}=\{e^{i\theta } : \theta \in [-\pi , \pi )\}$ is the one-dimensional torus. The Dirac measures $\delta _{0}$ and $\delta _{n}$ are $\delta _{n}(j)=0$ if $n\neq j$ and $\delta _{n}(n)=1$ for $n,j\in \mathbb{Z}$. Given a Banach space *X*, $X'$ is the dual of *X*, and ${\mathcal{B}}(X)$ is the set of linear bounded operators on *X*; given $A\in {\mathcal{B}}(X)$, we $A'\in {\mathcal{B}}(X')$ is the adjoint of the operator *A*. We denote by $\chi _{I}$ the indicator function of a set *I* (i.e., $\chi _{I}(n)= 1$ if $n\in I$ and $\chi _{I}(n)= 0$ if $n\notin I$). Furthermore, $I_{n}$ and $J_{n}$ are the Bessel functions. The usual set numbers $\mathbb{N}$, $\mathbb{N}_{0}=\mathbb{N}\cup \{0\}$, $\mathbb{Z}$, $\mathbb{R}$, and $\mathbb{C}$ are used. Furthermore, Γ is the gamma function, $\Phi _{\beta }$ is the Wright function (Sect. A.1), $E_{\alpha ,\beta }$ is the Mittag-Leffler function, $I_{n}$ and $J_{n}$ are the Bessel functions (Sect. A.2), and the stable Lévy distribution is denoted by $f_{t,\alpha }$ (Sect. A.3).

## A Banach algebra framework

Given $1\le p \le \infty $, we recall that the Banach spaces $(\ell ^{p}(\mathbb{Z}), \Vert \cdot \Vert _{p})$ are formed by biinfinite sequences $f=(f(n))_{n\in \mathbb{Z}}\subset \mathbb{C}$ such that

$$\begin{aligned}& \Vert f \Vert _{p}: = \Biggl(\sum_{n=-\infty }^{\infty } \bigl\vert f(n) \bigr\vert ^{p} \Biggr)^{\frac{1}{p}}< \infty , \quad 1\le p< \infty , \\& \Vert f \Vert _{\infty }: = \sup_{n\in \mathbb{Z}} \bigl\vert f(n) \bigr\vert < \infty . \end{aligned}$$

We recall the natural embeddings $\ell ^{1}(\mathbb{Z})\hookrightarrow \ell ^{p}(\mathbb{Z}) \hookrightarrow \ell ^{\infty }(\mathbb{Z})$ for $1\le p\le \infty $ and that the dual of $\ell ^{p}(\mathbb{Z})$ is identified with $\ell ^{p'}(\mathbb{Z})$, where $\frac{1}{p}+\frac{1}{p'}=1$ for $1< p<\infty $ and $p=1$ if $p'=\infty $.

In the case of $f\in \ell ^{1}(\mathbb{Z}) $ and $g\in \ell ^{p}(\mathbb{Z})$, we define

$$ (f\ast g) (n):=\sum_{j=-\infty }^{\infty }f(n-j)g(j), \quad n\in \mathbb{Z}. $$

From Young’s inequality it follows that $f\ast g\in \ell ^{p}(\mathbb{Z})$. Note that $(\ell ^{1}(\mathbb{Z}), \ast )$ is a commutative Banach algebra with identity $\delta _{0}:= \chi _{\{0\}}$. We observe that $\delta _{1}\ast \delta _{1}= \delta _{2}$ and, in general, $\delta _{n}\ast \delta _{m}=\delta _{n+m}$ for $n, m\in \mathbb{Z}$.

The Gelfand transform associated with $(\ell ^{1}(\mathbb{Z}), \ast )$ is the discrete Fourier transform $\mathcal{F}:\ell ^{1}(\mathbb{Z})\to C(\mathbb{T})$ (or Fourier series), where

$$\begin{aligned} \hat{f}(\theta ):=\mathcal{F}(f) \bigl(e^{i\theta }\bigr):=\sum _{n\in \mathbb{Z}}f(n)e^{in \theta }, \quad \theta \in \mathbb{T}. \end{aligned}$$

We recall that the spectrum of *f*, denoted $\sigma _{\ell ^{1}(\mathbb{Z})}(f)$, is defined by

$$ \sigma _{\ell ^{1}(\mathbb{Z})}(f):=\bigl\{ \lambda \in \mathbb{C} : (\lambda \delta _{0}-f)^{-1}\in \ell ^{1}(\mathbb{Z})\bigr\} . $$

In what follows, we consider the general theory of commutative Banach algebras as a framework. We collect the results that will be of our interest in the following theorem.

### Theorem 2.1

*The following properties hold*:

(i) *The spectrum* $\operatorname{Spec}(\ell ^{1}(\mathbb{Z}))$ *is compact and*, *consequently*, *homeomorphic to the unit complex circle* $\mathbb{T}:= \{z\in \mathbb{C}: |z|=1\}$.
(ii) $\sigma _{\ell ^{1}(\mathbb{Z})}(f)\subset \{z\in \mathbb{C} ; \vert z\vert <\Vert f\Vert _{1}\}$, *and*
  2.1 $$ (\lambda \delta _{0}-f)^{-1}=\sum _{n\ge 0}\lambda ^{-n-1}f^{n}, \quad \Vert f \Vert _{1}< \vert \lambda \vert . $$
(iii) *The algebra* $\ell ^{1}(\mathbb{Z})$ *is a semisimple regular Banach algebra*, *and the discrete Fourier transform* $\mathcal{F}$ *is injective*.
(iv) $\mathcal{F}(f\ast g)=\mathcal{F}(f)\mathcal{F}(g)$, *and*
  2.2 $$ \sigma _{\ell ^{1}(\mathbb{Z})}(f)= \mathcal{F}(f) (\mathbb{T}), \quad f \in \ell ^{1}(\mathbb{Z}). $$

### Proof

The first claim follows from the fact that the algebra $\ell ^{1}(\mathbb{Z})$ has an identity; see, for example, [35], and the second one can be found in [35, p. 116]. The proof of (ii) is straightforward. From [35, Theorem 4.7.4] it follows that $\ell ^{1}(\mathbb{Z})$ is semisimple and $\mathcal{F}$ is injective. By [35, Corolary 7.2.3] $\ell ^{1}(\mathbb{Z})$ is a regular Banach algebra. Statement (iv) is taken from [35, Theorem 3.4.1.]. □

We observe that the range of the Gelfand transform is the Wiener algebra ${\mathcal{A}}(\mathbb{T})$, the pointwise algebra of absolutely convergent Fourier series, that is, $F(e^{i \theta })= \sum_{n\in \mathbb{Z}} f(n) e^{i \theta n}$, $(\theta \in \mathbb{T})$ with $f\in \ell ^{1}(\mathbb{Z})$. For $F\in {\mathcal{A}}(\mathbb{T})$, we also write $F(z)=\sum_{n\in \mathbb{Z}} f(n) z^{n}$ for $\vert z\vert \le 1$.

The inverse discrete Fourier transform is given by the expressions

$$\begin{aligned} \mathcal{F}^{-1}(F) (n) = \frac{1}{2\pi } \int _{-\pi }^{\pi }F\bigl(e^{i \theta }\bigr) e^{-in\theta } \,d\theta = \frac{1}{2\pi i} \int _{|z|=1} F(z) \frac{dz }{z^{n+1}}, \quad n\in \mathbb{Z}, \end{aligned}$$

for $F\in {\mathcal{A}}(\mathbb{T})$ (and for other functions in larger sets).

The classical formulation of Wiener’s lemma characterizes the functions $F\in {\mathcal{A}}(\mathbb{T})$ that are invertible in ${\mathcal{A}}(\mathbb{T})$ as follows. For $F\in {\mathcal{A}}(\mathbb{T})$ where $F(e^{i\theta })=\sum_{n\in \mathbb{Z}} f(n) e^{i \theta n}$ for $\theta \in \mathbb{T}$, $F(e^{i\theta })\neq0$ for all $\theta \in \mathbb{T}$ if and only if $1/F \in {\mathcal{A}}(\mathbb{T})$, that is, $(1/F)(e^{i\theta })=\sum_{n\in \mathbb{Z}} g(n) e^{i \theta n}$ with $(g(n))_{n\in \mathbb{Z}}\in \ell ^{1}(\mathbb{Z})$; in this case, $f\ast g=\delta _{0} $ [32, Theorem 5.5].

Recall the definition of the classical Mittag-Leffler function (see (A.3)). We now introduce the following definition.

### Definition 2.2

For $\alpha , \beta >0$, we define the vector-valued Mittag-Leffler function $E_{\alpha ,\beta } : \ell ^{1}(\mathbb{Z})\to \ell ^{1}(\mathbb{Z})$, by

$$ E_{\alpha , \beta }(a) := \sum_{j=0}^{\infty } \frac{a^{ j}}{\Gamma (\alpha j + \beta )}, \quad a\in \ell ^{1}( \mathbb{Z}). $$

Note that

$$\begin{aligned} E_{1,1}(a)=\sum_{j=0}^{\infty } \frac{a^{ j}}{j!}=e^{a};\qquad E_{2,1}(a)= \sum _{j=0}^{\infty }\frac{a^{ j}}{(2j)!}. \end{aligned}$$

The set $\exp (\ell ^{1}(\mathbb{Z})):=\{e^{a} ;a \in \ell ^{1}( \mathbb{Z})\}$ is the connected component of $\delta _{0}$ in the set of regular elements in $\ell ^{1}(\mathbb{Z})$ [35, Theorem 6.4.1].

We follow the usual terminology in semigroup theory: the element *a* is called the generator of the entire group $(e^{z a})_{z\in \mathbb{C}}$; the cosine and sine functions are defined as $\operatorname{Cos}(z,a):=E_{2,1}(z^{2}a) $ and $\operatorname{Sin}(z,a):=zE_{2,2}(z^{2}a)$. We have

$$ \operatorname{Sin}(z,a)= \int _{[0,z]} \operatorname{Cos}(s,a)\,ds, \quad z\in \mathbb{C}, $$

for $a\in \ell ^{1}(\mathbb{Z})$; see [10, Sects. 3.1 and 3.14]. Moreover, the Laplace transform of an entire group or a cosine function is connected with the resolvent of its generator as follows:

2.3 $$\begin{aligned}& (\lambda -a)^{-1} = \int _{0}^{\infty }e^{-\lambda s} e^{as}\,ds, \quad \lambda >{ \Vert a \Vert _{1}}, \\& \lambda \bigl(\lambda ^{2}-a\bigr)^{-1} = \int _{0}^{\infty }e^{-\lambda s} \operatorname{Cos}(s,a) \,ds, \quad \lambda >\sqrt{ \Vert a \Vert _{1}}; \end{aligned}$$

see, for example, [10, p. 213].

### Example 2.3

For $\alpha , \beta >0$, we have

$$ E_{\alpha , \beta }(z\delta _{0})= E_{\alpha , \beta }(z)\delta _{0}; \qquad E_{\alpha , \beta }(z\delta _{1})=\sum _{j=0}^{\infty } \frac{z^{j}\delta _{j}}{\Gamma (\alpha j + \beta )}. $$

In particular, $e^{z\delta _{1}}={\sum_{j=0}^{\infty } \frac{z^{j}\delta _{j}}{j!}}$ and $\operatorname{Cos}(z, \delta _{1})={\sum_{j=0}^{ \infty }\frac{z^{2j}\delta _{j}}{(2j)!}}$ are generated by $\delta _{1}$.

Considering generalized versions of the Mittag-Leffler function, as well as of other hypergeometric series, as presented, for example, in [3–5], more examples can be easily derived.

In the next proposition, we collect some basic properties of these vector-valued Mittag-Leffler functions. As usual, we consider Bochner vector-valued integration in the Banach space $\ell ^{1}(\mathbb{Z})$; see, for example, [41, Sect. 1.2]. For the definition of the Wright function $\Phi _{\gamma }$, see the Appendix, formula (A.1).

### Proposition 2.4

*For* $\alpha , \beta >0$ *and* $a\in \ell ^{1}(\mathbb{Z})$, *we have*:

(i) $\Vert E_{\alpha , \beta }(a)\Vert _{1} \le E_{\alpha , \beta }(\Vert a \Vert _{1})$.
(ii) ${\mathcal{F}}( E_{\alpha , \beta }(a))= E_{\alpha , \beta }({\mathcal{F}}(a))$; *in particular*, ${\mathcal{F}}( e^{az})= e^{z{\mathcal{F}}(a)}$ *and* ${\mathcal{F}}( \operatorname{Cos}(z,a))= \operatorname{Cos}({{ \mathcal{F}}(z)},a)$ *for* $z\in \mathbb{C}$.
(iii) $\sigma _{\ell ^{1}(\mathbb{Z})}(E_{\alpha , \beta }(a))= E_{\alpha , \beta }(\sigma _{\ell ^{1}(\mathbb{Z})}(a))$.
(iv) *The following Laplace transform formula holds*:
  2.4 $$ \int _{0}^{\infty } e^{-\lambda t} t^{\alpha k + \beta -1} E_{\alpha , \beta }^{(k)}\bigl( t^{\alpha }a\bigr)\,dt ={k! \lambda ^{\alpha -\beta }} \bigl(\bigl( \lambda ^{\alpha } -a\bigr)^{-1} \bigr)^{(k+1)}, \quad {\Re }(\lambda )> \Vert a \Vert _{1}^{1/\alpha }, $$
  *for* $k\in \mathbb{N}\cup \{0\}$.
(v) *For* $0<\gamma <1$, $E_{\gamma ,1}(a)=\int _{0}^{\infty }\Phi _{\gamma }(t)e^{ta}\,dt$.

### Proof

Proofs of parts (i) and (ii) are straightforward. Part (iii) is the spectral mapping theorem shown in [35, Theorem 6.2.1]. Since the algebra $\ell ^{1}(\mathbb{Z})$ is semisimple (see Theorem 2.1), formulae in (iv) and (v) are direct consequences of the scalar identities [39, formula (180), p. 21]. □

Given $a\in \ell ^{1}(\mathbb{Z})$, the modified Mittag-Leffler function $S_{\alpha , \beta }: (0,\infty )\to \ell ^{1}(\mathbb{Z})$, which we define by

2.5 $$ S_{\alpha , \beta }(t,a):= t^{\beta -1}E_{\alpha , \beta } \bigl(t^{\alpha }a\bigr), \quad t>0,$$

is a $(g_{\alpha }, g_{\beta })$-regularized resolvent family generated by *a* in the algebra $\ell ^{1}(\mathbb{Z})$. For the definition of $(g_{\alpha }, g_{\beta })$-regularized resolvent families and more detail in the general case of linear and bounded operators in a Banach space, we refer the reader to [2, Sect. 4] and the survey [36].

We introduce the functions

$$ \psi _{\alpha ,\beta }(t,s):= t^{\beta -1}\sum_{n=0}^{\infty } \frac{(-st^{- \alpha })^{n}}{n!\Gamma (-\alpha n +\beta )}, \quad s,t>0, $$

for $0<\alpha <1$ and $\beta >0$. Note that $\psi _{\alpha ,1-\alpha }(s,t)=t^{-\alpha }\Phi _{\alpha }(st^{\alpha })$ for $0<\alpha <1$.

A direct consequence of [2, Theorem 12] is the following subordination theorem.

### Theorem 2.5

*Let* $0<\eta _{1}$, $0<{\eta _{2}}$, *and* $a\in \ell ^{1}(\mathbb{Z})$, *and let* $S_{\eta _{1},\eta _{2}}$ *be defined in* (2.5). *Then*

$$ S_{\alpha \eta _{1},\alpha \eta _{2}+\beta }(t,a)= \int _{0}^{\infty } \psi _{\alpha ,\beta }(t,s)S_{\eta _{1},\eta _{2}}(s,a) \,ds,\quad t>0, $$

*for* $0<\alpha <1$ *and* $\beta \ge 0$.

Note that in the case of $\eta _{1}=2$, $\eta _{2}=1$, and $\alpha =\beta =\frac{1}{2}$ in Theorem 2.5, we obtain the well-known relation between cosine and semigroup operators generated by *a*, known as the Weierstrass formula:

2.6 $$ e^{at}=\frac{1}{\sqrt{\pi t}} \int _{0}^{\infty }e^{-\frac{s^{2}}{4t}} \operatorname{Cos}(s,a) \,ds, \quad t>0, $$

for $a\in \ell ^{1}(\mathbb{Z})$; see, for example, [10, Theorem 3.14.17].

A nice application of the classical Wiener lemma is the invariance of spectrum for convolution operators defined on $\ell ^{p}(\mathbb{Z})$ for $1\le p\le \infty $. This issue is contained in the following theorem that is the key abstract result in this paper.

### Theorem 2.6

*For* $a\in \ell ^{1}(\mathbb{Z})$, *we define*

2.7 $$ A(b) (n):= (a\ast b) (n), \quad {n\in \mathbb{Z}}, b\in \ell ^{p}( \mathbb{Z}). $$

*Then* $A\in {\mathcal{B}}(\ell ^{p}(\mathbb{Z}))$ *for all* $1\le p\le \infty $. *Moreover*, $\Vert A\Vert =\Vert a\Vert _{1}$, *and for all* $1\le p\le \infty $, *we have the following identities*:

2.8 $$ \sigma _{{\mathcal{B}}(\ell ^{p}(\mathbb{Z}))}(A)=\sigma _{\ell ^{1}( \mathbb{Z})}(a)= \mathcal{F}(a) (\mathbb{T}). $$

*For all* $a\in \ell ^{1}(\mathbb{Z})$, *we have that* $e^{za}$ *is an entire group in* $\ell ^{p}(\mathbb{Z})$ *with generator* *a*, *and for all* $1\le p\le \infty $, *we have the following identities*:

2.9 $$ \sigma _{{\mathcal{B}}(\ell ^{p}(\mathbb{Z}))}\bigl(e^{za}\bigr)=\sigma _{\ell ^{1}( \mathbb{Z})}\bigl(e^{za}\bigr)=e^{z\mathcal{F}(a)(\mathbb{T})}, \quad z\in \mathbb{C}. $$

### Proof

From Young’s inequality it follows that $A\in {\mathcal{B}}(\ell ^{p}(\mathbb{Z}))$. Since the algebra $\ell ^{p}(\mathbb{Z})$ has the identity $\delta _{0}$, the property of the norm follows. For identities (2.8), we refer to [32, Corollary 5.20]. Finally, for the spectral mapping theorem (2.9), we use (2.8) and [35, Theorem 6.2.1]. □

The element *a* in the theorem is also called the *symbol* of the operator *A*.

### Remark 2.7

It is also straightforward to check that the adjoint operator of *A* is again a convolution operator given by $A'(g)(n):=(\tilde{a}\ast g)(n)$, where

$$ \tilde{a}(n)=a(-n), \quad n\in \mathbb{Z}. $$

## Some finite difference operators in $\ell ^{1}(\mathbb{Z})$

An important case of finite difference operators is given by sequences in the set

$$ c_{c}(\mathbb{Z}):=\bigl\{ a\in \ell ^{1}(\mathbb{Z}): \exists m \in \mathbb{Z}_{+}: a(n)=0, \forall \vert n \vert >m) \bigr\} . $$

In such a case, the discrete Fourier transform of $a \in c_{c}(\mathbb{Z})$ is the trigonometric polynomial

3.1 $$ \mathcal{F}(a) \bigl(e^{i\theta }\bigr)=\sum _{j=-m}^{m}a(j)e^{ij\theta }. $$

It is interesting to observe that if $\sum_{j=-m}^{m}a(j)=0$, then $0\in \sigma _{\ell ^{1}(\mathbb{Z})}(a)$. This immediately follows from (2.8).

In this paper, we concentrate our study on the operators that appear in the seminal paper of Bateman [16].

### Definition 3.1

For $f\in \ell ^{p}(\mathbb{Z})$ with $1\le p\le \infty $, we define the following operators:

1. $-\Delta f(n):=f(n)-f(n+1)=((\delta _{0}-\delta _{-1})\ast f)(n)$;
2. $\nabla f(n):=f(n)-f(n-1)=((\delta _{0}-\delta _{1})\ast f)(n)$;
3. $\Delta _{d}f(n):=f(n+1)-2f(n)+f(n-1)=((\delta _{-1}-2\delta _{0}+ \delta _{1})\ast f)(n)$; and
4. $\Delta _{dd}f(n):=f(n+2)-2f(n)+f(n-2)=((\delta _{-2}-2\delta _{0}+ \delta _{2})\ast f)(n)$

for $n\in \mathbb{Z}$.

We remark that when considering the above-defined operators in the context of numerical analysis, the operators −Δ and ∇ are related to the Euler scheme of approximation, and the operator $\Delta _{d}$ corresponds to the second-order central difference approximation for the second-order derivative. The operator $\Delta _{dd}$ appears in Bateman’s paper [16, p. 506] in connection with the equations of Born and Karman on crystal lattices in vibration.

### The operator −Δ

The forward difference operator $\Delta f(n):=f(n+1)-f(n)$ is a classical operator used in approximation theory and in the theory of difference equations. Considering it as an operator from $\ell ^{p}(\mathbb{Z})$ to $\ell ^{p}(\mathbb{Z})$, our main result is as follows.

#### Theorem 3.2

*The operator* $-\Delta f = a\ast f$, *where* $a := \delta _{0} -\delta _{-1}$, *possesses the following properties*:

1. *The norm is given by* $\|\Delta \|=2$;
2. *The Fourier transform is* $\mathcal{F}(a)(z)=1-z$, $|z|=1 $;
3. *For all* $1\leq p \leq \infty $, *the spectrum is given by* $\sigma _{{\mathcal{B}}(\ell ^{p}(\mathbb{Z}))}(-\Delta ) = \{z\in \mathbb{T} : \vert z-1\vert =1\}$;
4. *For* $\vert \lambda +1\vert >1$,
  $$ (\lambda \delta _{0}+a)^{-1}=\sum _{j\ge 0}\frac{\delta _{-j}}{(1+ \lambda )^{j+1}}. $$
5. *The associated group is* $e^{-za}(n)= e^{-z}\frac{z^{-n}}{(-n)!}\chi _{- \mathbb{N}_{0}}(n)$, $z\in \mathbb{C}$, $n\in \mathbb{Z}$, *and its generator is* −*a*.
6. *The norm of the group is given by* $\|e^{-ta}\|_{1}=1$, $t>0$;
7. *The associated cosine function is* $\operatorname{Cos}(z,-a)(n)= \frac{\sqrt{\pi } }{{(-n)!}} (\frac{z }{2} )^{-n+\frac{1}{2}}{J_{-n-\frac{1}{2}}(z)}\chi _{- \mathbb{N}_{0}}(n)$ *for* $z\in \mathbb{C}$ *and* $n\in \mathbb{Z} $.

#### Proof

(1) The Minkowski inequality shows that $\|\Delta \|\leq 2$. Then observe that $\delta _{0} \in \ell ^{p}(\mathbb{Z})$ with $\|\delta _{0}\|_{p}=1$ satisfies $\|\Delta \delta _{0}\|_{p}=2$, proving the claim. (2) Follows immediately from the definition of the discrete Fourier transform. (3) Follows from formula (2.8) in Theorem 2.6 and (2).

To prove (4), we apply (2.1) to get

$$ (\lambda \delta _{0}+a)^{-1}=\bigl((\lambda +1)\delta _{0}-\delta _{-1}\bigr)^{-1}= \sum _{j\ge 0}\frac{\delta _{-j}}{(1+\lambda )^{j+1}} $$

for $\vert \lambda +1\vert >1$. We show (5) directly:

$$ e^{-za}(n)= \bigl(e^{z\delta _{-1}}\ast e^{-z\delta _{0}} \bigr) (n)= \bigl(e^{z\delta _{-1}}\ast e^{-z}\delta _{0} \bigr) (n)=e^{-z} \frac{z^{-n}}{(-n)!}\chi _{-\mathbb{N}_{0}}(n) $$

for $z\in \mathbb{C}$ and $n\in \mathbb{Z}$. The norm $\|e^{-ta}\|_{1}=1$ for $t>0$ is straightforward from (5). Finally, to show (7), we apply the Laplace transform and formula (A.10) to get

$$\begin{aligned} \frac{\sqrt{\pi } }{{(-n)!}} \int _{0}^{\infty }e^{-\lambda t} \biggl( \frac{t }{2} \biggr)^{-n+\frac{1}{2}}J_{-n-\frac{1}{2}}(t)\,dt= \frac{\lambda }{(\lambda ^{2}+1)^{-n+1}},\quad \lambda >1, \end{aligned}$$

for $n\le 0$. By (4) we have that

$$ \frac{\lambda }{(\lambda ^{2}+1)^{-n+1}}= {\lambda \bigl(\lambda ^{2}+a \bigr)^{-1}(n)}, \quad n\le 0, $$

and we apply (2.3) to conclude the claimed equality and identify the generator of the cosine function with −*a*. □

We remark that groups generated by Δ are treated in [1, Sect. 2] and cosine functions in [16, Introduction].

### The operator ∇

This operator corresponds to the classical backward difference operator.

#### Theorem 3.3

*The operator* $\nabla f = a\ast f$, *where* $a:= \delta _{0}-\delta _{1}$, *possesses the following properties*:

1. $\|\nabla \|=2$;
2. $\mathcal{F}(a)(z)= 1-\frac{1}{z}$;
3. *For all* $1\leq p \leq \infty $, *we have* $\sigma _{{\mathcal{B}}(\ell ^{p}(\mathbb{Z}))}(\nabla ) = \{z\in \mathbb{T} : \vert z-1\vert =1\}$;
4. *For* $\vert \lambda +1\vert >1$,
  $$ (\lambda \delta _{0}+a)^{-1}=\sum _{j\ge 0}\frac{\delta _{j}}{(1+ \lambda )^{j+1}}. $$
5. $e^{-za}(n)= e^{-z}\frac{z^{n}}{n!}\chi _{\mathbb{N}_{0}}(n)$, $z \in \mathbb{C}$, $n\in \mathbb{Z}$;
6. $\|e^{-ta}\|_{1}=1$, $t>0$;
7. $\operatorname{Cos}(z,-a)= \frac{\sqrt{\pi } }{{n!}} (\frac{z}{2} )^{n+\frac{1}{2}}{J_{n-\frac{1}{2}}(z)}\chi _{\mathbb{N}_{0}}(n)$, $z\in \mathbb{C}$, $n\in \mathbb{Z}$.

#### Proof

The proofs of statements (1), (2), (3), and (4) follow the lines of Theorem 3.2. For statement (5), we have

$$ e^{-za}(n)= \bigl(e^{z\delta _{1}}\ast e^{-z}\delta _{0} \bigr) (n)=e^{-z} \frac{z^{n}}{n!}\chi _{\mathbb{N}_{0}}(n) $$

for $z\in \mathbb{C}$ and $n\in \mathbb{Z}$. Claim (6) follows from (5). Finally, we check (7) as follows. We apply Laplace transform and formula (A.10) to get

$$\begin{aligned} \frac{\sqrt{\pi } }{{n!}} \int _{0}^{\infty }e^{-\lambda t} \biggl( \frac{t }{2} \biggr)^{n+\frac{1}{2}}J_{n-\frac{1}{2}}(t)\,dt= \frac{\lambda }{( \lambda ^{2}+1)^{n+1}},\quad \lambda >1, \end{aligned}$$

for $n\ge 0$. Then we can apply (4), (2.3), and the uniqueness of the Laplace transform to conclude (7). □

We remark that groups generated by −∇ are treated in [1, Sect. 2] and cosine functions in [16, Introduction].

We observe that when considering the Fourier transform in the context of signal processing, the conversion from continuous-time systems to discrete-time systems is done through the Euler transformation $1-\frac{1}{z}$. In such a context, it is important to remark that $z^{-1}$ represents a delay in time.

### The operator $\Delta _{d}$

#### Theorem 3.4

*The operator* $\Delta _{d} f = a\ast f$, *where* $a:= \delta _{-1} -2\delta _{0} +\delta _{1}$, *possesses the following properties*:

1. $\|\Delta _{d} \|=4$;
2. $\mathcal{F}(a)(z)= z+\frac{1}{z} -2$;
3. *For all* $1\leq p \leq \infty $, *we have* $\sigma _{{\mathcal{B}}(\ell ^{p}(\mathbb{Z}))}(\Delta _{d}) = [-4,0]$;
4. *The group* $e^{za}(n)= e^{-2z}I_{n}(2z)$, $z\in \mathbb{C}$, $n\in \mathbb{Z}$, *and its generator is* *a*;
5. $\|e^{ta}\|_{1}=1$, $t>0$;
6. *For* $\lambda \in \mathbb{C}\setminus [-4,0]$,
  $$ (\lambda -a)^{-1}(n)= 2^{-n}\frac{((\lambda +2)-\sqrt{\lambda ^{2}+4 \lambda })^{n}}{\sqrt{\lambda ^{2}+4\lambda }}, \quad n\in \mathbb{Z}; $$
7. $\operatorname{Cos}(z,a)= J_{2n}(2z)$, $z\in \mathbb{C}$, $n \in \mathbb{Z} $.

#### Proof

Statements (1) and (2) follow as in the previous theorems. To prove (3), observe that

$$\begin{aligned} \sigma _{{\mathcal{B}}(\ell ^{p}(\mathbb{Z}))}(\Delta _{d}) &= \biggl\{ z \in \mathbb{C} : z= w+\frac{1}{w} -2, \vert w \vert =1 \biggr\} = \bigl\{ z\in \mathbb{C} : z= 2\bigl(\Re (w)-1\bigr), \vert w \vert =1 \bigr\} \\ &= \bigl\{ z\in \mathbb{C} : z= 2\bigl(\cos (\theta ) -1\bigr), \theta \in [0,2 \pi ) \bigr\} =[-4,0]. \end{aligned}$$

To show (4), we proceed as in the previous theorems, obtaining

$$\begin{aligned} e^{za}(n) &= \bigl(\bigl(e^{z\delta _{1}}\ast e^{z\delta _{-1}}\bigr) \ast e^{-2z} \delta _{0} \bigr) (n)= e^{-2z} \bigl(e^{z\delta _{1}}\ast e^{z\delta _{-1}}\bigr) (n) \\ &= e^{-2z}\sum_{j=-\infty }^{\infty } \frac{z^{n-j}}{(n-j)!}\chi _{ \mathbb{N}_{0}}(n-j) \frac{z^{-j}}{(-j)!}\chi _{-\mathbb{N}_{0}}(j) \\ &=e^{-2z} \sum_{j=0}^{\infty } \frac{z^{n+j}}{(n+j)!}\frac{z^{j}}{j!} = e^{-2z} I_{n}(2z), \end{aligned}$$

where we have used (A.7) in the last identity. To prove (5), we use (4) and Appendix A.2(4). To prove (6), we apply the Laplace transform and formula (A.8) to get

$$\begin{aligned} (\lambda -a)^{-1}(n) =& \int _{0}^{\infty }e^{-\lambda t}e^{t a}(n) \,dt= \int _{0}^{\infty }e^{-(\lambda +2) t}I_{n}(2t) \,dt=2^{-n}\frac{((\lambda +2)- \sqrt{\lambda ^{2}+4\lambda })^{n}}{\sqrt{\lambda ^{2}+4\lambda }} \end{aligned}$$

for $\Re \lambda >0$ and $n\in \mathbb{Z}$. By the principle of analytic continuation we can extend the equality to the set $\lambda \in \mathbb{C}\setminus [-4,0]$. Finally, to show (7), we apply again the Laplace transform and formula (A.9) to get

$$ \int _{0}^{\infty }e^{-\lambda t}J_{2n}(2t) \,dt= 2^{-2n}\frac{ (\sqrt{ \lambda ^{2}+4}-\lambda )^{2n}}{\sqrt{\lambda ^{2}+4}}= 2^{-n}\frac{ (\lambda ^{2}+2-\lambda \sqrt{\lambda ^{2}+4} )^{n}}{\sqrt{\lambda ^{2}+4}}= \lambda \bigl(\lambda ^{2}-a\bigr)^{-1}(n), $$

where we have applied (6) for $\Re \lambda >0$ and $n\in \mathbb{Z}$. □

We observe that groups generated by the discrete Laplacian $\Delta _{d}$ are treated in [21, Sect. 2] and cosine functions in [37, Theorem 1.2]. Here we have presented a complete and alternative approach using the framework of Banach algebras combined with the Laplace transform method.

### The operator $\Delta _{dd}$

#### Theorem 3.5

*The operator* $\Delta _{dd} f = a\ast f$, *where* $a:= \delta _{-2} -2\delta _{0} +\delta _{2}$, *possesses the following properties*:

1. $\|\Delta _{dd} \|=4$;
2. $\mathcal{F}(a)(z)= (z-\frac{1}{z})^{2}$;
3. *For all* $1\leq p \leq \infty $, *we have* $\sigma _{{\mathcal{B}}(\ell ^{p}(\mathbb{Z}))}(\Delta _{dd}) = [-4,0]$;
4. $e^{za}(n)= e^{-2z}I_{\frac{n}{2}}(2z)\chi _{2\mathbb{Z}}(n)$, $z\in \mathbb{C}$, $n\in \mathbb{Z}$;
5. $\|e^{-ta}\|_{1}=1$, $t>0$;
6. *For* $\lambda \in \mathbb{C}\setminus [-4,0]$,
  $$ (\lambda -a)^{-1}(n)= 2^{-\frac{n}{2}}\frac{((\lambda +2)-\sqrt{\lambda ^{2}+4 \lambda })^{\frac{n}{2}}}{\sqrt{\lambda ^{2}+4\lambda }}\chi _{2 \mathbb{Z}}(n), \quad n\in \mathbb{Z}; $$
7. $\operatorname{Cos}(z,-a)(n)= J_{n}(2z)\chi _{2\mathbb{Z}}(n)$, $z \in \mathbb{C}$, $n\in \mathbb{Z}$.

#### Proof

En view of the previous theorems, (1) and (2) are straightforward. To prove (3), observe that

$$\begin{aligned} \sigma _{{\mathcal{B}}(\ell ^{p}(\mathbb{Z}))}(\Delta _{dd}) &= \bigl\{ z \in \mathbb{C} : z= -4\sin ^{2} \theta , \theta \in [0,2 \pi ) \bigr\} =[-4,0]. \end{aligned}$$

We show (4): To show (4), we apply the discrete Fourier transform and Theorem 3.4 ((2) and (4)) to get

$$ {\mathcal{F}}\bigl( e^{-2t}I_{\frac{n}{2}}(2t)\chi _{2\mathbb{Z}}(n) \bigr) (z)=\sum_{j \in \mathbb{Z}} e^{-2t}I_{j}(2t) \bigl(z^{2}\bigr)^{ j}={\mathcal{F}}\bigl(e^{t \Delta _{d}} \bigr) \bigl(z^{2}\bigr)=e^{t(z-\frac{1}{z})^{2}}={\mathcal{F}} \bigl(e^{ta}\bigr) (z) $$

for $z\in \mathbb{T}$, and we conclude equality (4) by the uniqueness of the discrete Fourier transform. The equality in (5) is a consequence of (4) and Appendix A.2(4). The proofs of (6) and (7) are similar to those of (6) and (7) in Theorem 3.4. □

Some simple computations show linear, algebraic, and dual relations between the operators defined previously, which are presented in the following result.

#### Proposition 3.6

*The discrete operators* −Δ, ∇, $\Delta _{d}$, *and* $\Delta _{dd}$ *possess the following properties*:

(i) *We have the equalities*
  $$ -\Delta _{d}=(\nabla - \Delta )= -\Delta \nabla ; $$
(ii) *For* $1\le p<\infty $, *we have the following identities on* $\ell ^{p}(\mathbb{Z})$:
  $$\begin{aligned} &(-\Delta )'=\nabla ; \qquad (\nabla )'=-\Delta ; \\ &(\Delta _{d})'=\Delta _{d}; \qquad (\Delta _{dd})'=\Delta _{dd}. \end{aligned}$$

#### Proof

The proofs are straightforward and left to the reader. □

In the next theorem, we present a decomposition for the Bessel function, which seems to be new. For simplicity, for $n\in \mathbb{Z}$ and $z\in \mathbb{C}$, we define

$$ g_{z,-}(n):=\frac{z^{n}}{n!}\chi _{\mathbb{N}_{0}}(n),\qquad g_{z,+}(n):= \frac{z^{-n}}{(-n)!}\chi _{-\mathbb{N}_{0}}(n). $$

#### Theorem 3.7

*The Bessel function* $I_{n}$ *admits a factorization via convolution given by*

$$ I_{n}(2z)=(g_{z,+}\ast g_{z,-}) (n), \quad n\in \mathbb{Z}, z\in \mathbb{C}. $$

#### Proof

We apply (4) in Theorem 3.4, Proposition 3.6(i), and Theorems 3.2 and 3.3(5), to get

$$ e^{-2z}I_{n}(2z)=e^{\Delta _{d} z}(n)=e^{-z(-\Delta )}e^{-t\nabla }(n)= e^{-2z} (g_{z,+}\ast g_{z,-}) (n) $$

for $n\in \mathbb{Z}$ and $z \in \mathbb{C}$. □

## Fractional powers of generators of uniformly bounded semigroups in $\ell ^{1}(\mathbb{Z})$

As we have commented in the introduction, to define fractional powers in a Banach algebra (and in operator theory) is, in general, a difficult task. Not every element in $\ell ^{1}(\mathbb{Z})$ has fractional powers. For example, $\delta _{1}$ does not have square root in $\ell ^{1}(\mathbb{Z})$. In contrast, there may be a continuous function $f\in C(\mathbb{T})$ such that $(f(z))^{2}=z$ for $z\in \mathbb{T}$.

When $\sigma _{\ell ^{1}(\mathbb{Z})}(a)\subset \mathbb{C}^{+}$ and $\alpha \in \mathbb{R}$, we may consider the function $F_{\alpha }(z)=z^{\alpha }$, which is holomorphic in a neighborhood of $\sigma _{\ell ^{1}(\mathbb{Z})}(a)$. By the analytic functional calculus, the element

$$ F_{\alpha }(a)=\frac{1}{2 \pi i} \int _{\gamma }\frac{F_{\alpha }(z)}{z-a}\,dz, $$

(where *γ* is a spectral contour lying in an open set ${\mathcal{O}}$ containing the spectrum of *a*) exists in the Banach algebra $\ell ^{1}(\mathbb{Z})$, and ${\mathcal{F}}(F_{\alpha }(a))=({\mathcal{F}}(a))^{\alpha }$ [35, Lemma 6.1.2]. Then $F_{\alpha }(a)$ is a fractional power of *a* of order *α*, and we write $F_{\alpha }(a)=a^{ \alpha }$. Note that there exists a classic way to define fractional powers of generators of uniformly bounded semigroups in Banach spaces; see, for example, [47, p. 260–264] and [33, Example 3.4.6-7].

As the next definition shows, we may follow a general methodology to treat fractional powers of elements in $\ell ^{1}(\mathbb{Z})$, analogously to the case of operators in Banach spaces; see [47, p. 265].

### Definition 4.1

Let $0<\alpha <1$, and let $a \in \ell ^{1}(\mathbb{Z})$ be such that $(e^{ta})_{t\ge 0}$ is a uniformly bounded semigroup, that is, $\sup_{s>0}\Vert e^{as}\Vert _{1}<\infty $. Then we write by $(-a)^{\alpha }$ the fractional power of *a* given by the following integral representation:

$$ (-a)^{\alpha }:=\frac{1}{\Gamma (-\alpha )} \int _{0}^{\infty }\frac{e^{sa}- \delta _{0}}{s^{1+\alpha }} \,ds. $$

### Remark 4.2

In fact, Definition 4.1 is an analogous formula in $\ell ^{1}(\mathbb{Z})$ of the well-known equality

$$ z^{\alpha }=\frac{1}{\Gamma (-\alpha )} \int _{0}^{\infty }\frac{e^{-zt}-1 }{t^{1+\alpha }}\,dt, \quad \Re z>0. $$

As an immediate consequence of this definition, we have that for $0<\alpha <1$,

4.1 $$ {\mathcal{F}}\bigl((-a)^{ \alpha }\bigr)=\bigl(-{ \mathcal{F}(a)}\bigr)^{ \alpha }, \qquad \sigma \bigl((-a)^{\alpha } \bigr)=\bigl(\sigma (-a)\bigr)^{ \alpha }, $$

where we have applied (2.8).

It is well known that the uniformly bounded semigroup $(e^{-t(-a)^{\alpha }})_{t\ge 0}$ is subordinated to $(e^{ta})_{t\ge 0}$ (principle of Lévy subordination) by the formula

4.2 $$ e^{-t(-a)^{\alpha }}= \int _{0}^{\infty }f_{t, \alpha }(s)e^{a s} \,ds=\sum_{j=0}^{\infty }\frac{(-t)^{j}}{j!}(-a)^{j\alpha }, \quad t\ge 0; $$

see, for example, [47, Theorem 1, p. 263]. Note that

$$ {\mathcal{F}}\bigl(e^{-t(-a)^{ \alpha }}\bigr)=e^{-t(-{\mathcal{F}(a)})^{ \alpha }}. $$

Now we present the fractional powers of the four elements in $\ell ^{1}(\mathbb{Z})$ given in Definition 3.1. For $a\in \ell ^{1}(\mathbb{Z})$, note that $A\in {\mathcal{B}}(\ell ^{p}(\mathbb{Z}))$, where $A(f):= a\ast f$ for $f\in \ell ^{p}(\mathbb{Z})$ and $1\le p\le \infty $. In the case that the fractional power $a^{\alpha }\in \ell ^{1}(\mathbb{Z})$ for $\Re \alpha >0$, we have $A^{\alpha }\in {\mathcal{B}}(\ell ^{p}(\mathbb{Z}))$, where $A^{\alpha }(f):= a^{\alpha }\ast f $ for $f\in \ell ^{p}(\mathbb{Z})$ and $1\le p\le \infty $.

### The operators $(-\Delta )^{\alpha }$ and $(\nabla )^{\alpha }$

We define the sequence

$$ k^{\alpha }(j):= \frac{\Gamma (\alpha +n)}{\Gamma (\alpha ) n!} = (-1)^{n}\binom{- \alpha }{n}, \quad n \in \mathbb{N}_{0}, $$

for $\alpha \in \mathbb{C} \setminus \{ 0,-1,-2,\ldots\}$; see [27, Sect. 2] and references therein. The sequence $k^{\alpha }$ satisfies the following identity [27, Proposition 3.1]:

4.3 $$ \sum_{j=0}^{\infty } k^{\alpha }(j)z^{j} = \frac{1}{(1-z)^{\alpha }}, \quad z\in \mathbb{C}, \vert z \vert \leq 1, z\neq 1. $$

The sequence $k^{\alpha }$ has been deeply investigated in several references; see, for example, [49, Vol. I, p. 77], where $A_{n}^{\alpha -1}= k^{\alpha }(n)$. For $0<\alpha <1$, note that $k^{-\alpha }(0)=1 $ and

$$ \sum_{j=0}^{\infty }k^{-\alpha }(j)=0, \qquad \sum_{j=1}^{\infty }k^{- \alpha }(j)=-1; $$

see also [1, Sect. 2], where the notation $\Lambda ^{\alpha } \equiv k^{-\alpha }$ is employed. For *j* large enough, $k^{-\alpha }(j)$ is of constant sign [49, Theorem 1.17] for $\alpha >0$; in particular, for $0<\alpha <1$, $k^{-\alpha }(j)<0$ for $j\in \mathbb{N}$.

Since $-\Delta (f):= (\delta _{0}-\delta _{-1})\ast f$ and $\nabla (f):= (\delta _{0}-\delta _{1})\ast f$ for $f\in \ell ^{p}(\mathbb{Z})$, we denote

4.4 $$ K_{+}^{\alpha }:=(\delta _{0}-\delta _{-1})^{\alpha },\qquad K_{-}^{\alpha }:=(\delta _{0}-\delta _{1})^{\alpha }. $$

We have the following result concerning properties of the kernels $K_{+}^{\alpha }$ and $K_{-}^{\alpha }$.

#### Theorem 4.3

*For all* $0<\alpha <1$, *we have*

4.5 $$ {\mathcal{F}}\bigl(K_{+}^{\alpha }\bigr) (z)= \biggl(1-\frac{1}{z}\biggr)^{\alpha } \quad \textit{and}\quad { \mathcal{F}}\bigl(K_{-}^{\alpha }\bigr) (z)=(1-{ z})^{\alpha } $$

*for* $z\in \mathbb{T}$ *and*

4.6 $$ K_{+}^{\alpha }= \sum _{j=0}^{\infty }k^{-\alpha }(j)\delta _{- j}, \qquad K_{-}^{\alpha }= \sum_{j=0}^{\infty }k^{-\alpha }(j) \delta _{ j}. $$

*In particular*, $\sigma (K_{+}^{\alpha })= \sigma (K_{-}^{\alpha })=\{(1-e^{i\theta })^{\alpha }\mid \theta \in [-\pi , \pi )\}$. *Moreover*,

$$ \bigl\Vert K^{\alpha }_{+} \bigr\Vert _{1}= \bigl\Vert K^{\alpha }_{-} \bigr\Vert _{1}=2 $$

*for* $0<\alpha <1$.

#### Proof

Using the first identity in (4.1), we obtain

$$ {\mathcal{F}}\bigl(K_{+}^{\alpha }\bigr) (z)= \bigl[{ \mathcal{F}}(K_{+})\bigr]^{\alpha }(z) = \Biggl[\sum _{j=0}^{\infty } (\delta _{0} -\delta _{-1}) (j) z^{j} \Biggr]^{ \alpha } = \biggl(1- \frac{1}{z}\biggr)^{\alpha }, $$

proving the first identity in (4.5). On the other hand, note that $[\sum_{j=0}^{\infty }k^{-\alpha }(j)\delta _{- j} ](n)= k^{- \alpha }(-n)$. Therefore, taking into account (4.3), we obtain

$$ {\mathcal{F}}\Biggl( \Biggl[\sum_{j=0}^{\infty }k^{-\alpha }(j) \delta _{- j} \Biggr]\Biggr) (z)={ \mathcal{F}}\bigl(k^{-\alpha }(- \cdot )\bigr) (z)=\biggl(1-\frac{1}{z}\biggr)^{\alpha }. $$

Then by the uniqueness of the Fourier transform we conclude the first identity in (4.6). The proof of the second identities in (4.5) and (4.6) is analogous. The property of the spectrum follows from the second identity in (4.1). Finally, we have that

$$ \bigl\Vert K^{\alpha }_{+} \bigr\Vert _{1}= \sum _{j=0}^{\infty } \bigl\vert k^{-\alpha }(j) \bigr\vert = 1- \sum_{j=1}^{\infty }k^{-\alpha }(j)=2, $$

and we conclude the proof. □

Now we consider the groups generated by the fractional powers $K^{\alpha }_{+}$ and $K^{\alpha }_{-}$ as elements of the Banach algebra $\ell ^{1}(\mathbb{Z})$.

#### Theorem 4.4

*For* $0<\alpha <1$ *and* $z\in \mathbb{C}$, *we have*

$$\begin{aligned}& e^{zK^{\alpha }_{+}} = e^{z}\delta _{0}+\sum _{j=1}^{\infty } \Biggl( \sum_{n=1}^{\infty } \frac{z^{n}}{n!}k^{-\alpha n}(j) \Biggr)\delta _{- j}, \\& e^{zK^{\alpha }_{-}} = e^{z}\delta _{0}+\sum _{j=1}^{\infty } \Biggl(\sum_{n=1}^{\infty } \frac{z^{n}}{n!}k^{-\alpha n}(j) \Biggr)\delta _{ j}. \end{aligned}$$

*In particular*, *for* $z\in \mathbb{C}$ *and* $w\in \mathbb{T}$, *we have*

$$ {\mathcal{F}}\bigl(e^{zK^{\alpha }_{+}}\bigr) (w)=e^{z(1-\frac{1}{w})^{\alpha }}, \qquad { \mathcal{F}}\bigl(e^{zK^{\alpha }_{-}}\bigr) (w)=e^{z(1-{ w})^{\alpha }}, $$

*and* $\sigma (e^{zK^{\alpha }_{+}})=\sigma (e^{zK^{\alpha }_{-}})=\{e^{z(1-{ e^{i \theta }})^{\alpha }}\mid \theta \in [-\pi , \pi )\} $.

*Moreover*, *the semigroups* $(e^{-tK^{\alpha }_{+}})_{t\ge 0}$ *and* $(e^{-tK^{\alpha }_{-}})_{t\ge 0}$ *are uniformly bounded*, *and*

$$ e^{-t}+\sum_{j=1}^{\infty } \Biggl\vert \sum_{n=1}^{\infty }\frac{(-t)^{n}}{n!}k^{- \alpha n}(j) \Biggr\vert \le 1, \quad t>0. $$

#### Proof

Note that

$$\begin{aligned} e^{zK^{\alpha }_{+}} =&\delta _{0}+\sum_{n=1}^{\infty } \frac{z^{n}}{n!}( \delta _{0}-\delta _{-1})^{n\alpha }= \delta _{0}+\sum_{n=1}^{\infty } \frac{z^{n} }{n!}\sum_{j=0}^{\infty }k^{-\alpha n}(j) \delta _{-j} \\ =&e^{z}\delta _{0}+\sum_{j=1}^{\infty } \Biggl( \sum_{n=1}^{\infty }\frac{z^{n} }{n!}k^{-\alpha n}(j) \Biggr)\delta _{- j} \end{aligned}$$

for $z\in \mathbb{C}$.

In the case $0<\alpha <1$, since $-(\delta _{0}-\delta _{-1})$ and $-(\delta _{0}-\delta _{1})$ generate uniformly bounded semigroups, the fractional powers $-K^{\alpha }_{+}$ and $-K^{\alpha }_{-}$ also generate uniformly bounded semigroups. Then

$$\begin{aligned} e^{-t}+\sum_{j=1}^{\infty } \Biggl\vert \sum_{n=1}^{\infty }\frac{(-t)^{n} }{n!}k^{-\alpha n}(j) \Biggr\vert = & \bigl\Vert e^{-tK_{+}^{\alpha }} \bigr\Vert _{1} \\ \le& \int _{0}^{\infty }f_{t, \alpha }(s) \bigl\Vert e^{-( \delta _{0}-\delta _{-1}) s} \bigr\Vert _{1} \,ds\le \int _{0}^{\infty }f_{t, \alpha }(s) \,ds=1 \end{aligned}$$

for $t\ge 0$, where we have applied [47, Proposition 3, p. 262]. □

Now we apply the Lévy subordination principle (4.2) to $a= \delta _{-1}-\delta _{0}$ or $a= \delta _{1}-\delta _{0}$ and by Theorem 4.4 obtain the following result. Note that this formula is also obtained from (A.12).

#### Corollary 4.5

*Let* $0<\alpha <1$, *and let* $f_{s,\alpha }$ *be the Lévy stable process defined by* (A.11). *Then*

$$ \sum_{j=1}^{\infty } k^{-\alpha j}(n) \frac{(-t)^{j}}{j!} = \int _{0}^{ \infty } f_{t,\alpha }(s) \frac{e^{-s}s^{n}}{n!}\,ds, \quad t>0, n\ge 1.$$

*In particular*, *when* $\alpha =\frac{1}{2}$, *we have*

$$ \sum_{j=1}^{\infty } k^{\frac{- j}{2}}(n) \frac{(-t)^{j}}{j!} = \int _{0}^{ \infty } \frac{t}{\sqrt{4\pi s^{3}}}e^{\frac{-t^{2}}{4s}} e^{-s}s^{n}\,ds, \quad n\ge 1. $$

### The operator $(-\Delta _{d})^{\alpha }$

The operator $(-\Delta _{d})^{\alpha }$ for $0<\alpha \le 1$, called the fractional discrete Laplacian, has been deeply treated in [22, 23, 26, 37]. In [37, Sect. 3] the sequence $(-\delta _{-1}+2\delta _{0}-\delta _{1})^{\alpha }$ is denoted by $K^{\alpha }_{d}$. To follow the notation in that paper, we write

$$ K^{\alpha }_{d}(n):=\frac{1}{2\pi } \int _{-\pi }^{\pi }\bigl(4\sin ^{2}(\theta /2)\bigr)^{\alpha }e^{-in\theta }\,d\theta = \frac{(-1)^{n}\Gamma (2\alpha +1)}{\Gamma (1+\alpha +n)\Gamma (1+\alpha -n)} $$

for $n\in \mathbb{Z}$ and $\alpha >0$ [37, Formula (22)]. In the case $1+\alpha +n\in -\mathbb{N}_{0}$, $K^{\alpha }_{d}(n)=0$. Then $\vert K^{\alpha }_{d}(n)\vert \sim \frac{\Gamma (2\alpha +1)}{\pi } \vert n\vert ^{-2\alpha -1}$ as $n\to \pm \infty $.

We summarize the main properties of the kernel $K^{\alpha }_{d}$ in the following result.

#### Theorem 4.6

*For* $0<\alpha <1$, *we have*

4.7 $$ {\mathcal{F}} \bigl(K^{\alpha }_{d}\bigr) (z)= \biggl(2-\biggl(z+\frac{1}{z}\biggr)\biggr)^{\alpha }= \biggl(4 \sin ^{2}\biggl(\frac{\theta }{2}\biggr)\biggr)^{\alpha }, \quad z=e^{i\theta }\in \mathbb{T},$$

*and*

4.8 $$ K_{d}^{\alpha }=K_{+}^{\alpha } \ast K_{-}^{\alpha }. $$

*In particular*,

4.9 $$ K^{\alpha }_{d} = \sum _{j=0}^{\infty } \bigl(k^{-\alpha }_{-} * k^{-\alpha }\bigr) (j) \delta _{j}, $$

*where* $k^{-\alpha }_{-}(n):=k^{-\alpha }(-n)$. *Moreover*, $\sigma (K^{\alpha }_{d})=[0, 4^{\alpha }]$, *and*

$$ \bigl\Vert K^{\alpha }_{d} \bigr\Vert _{1}=2 \frac{\Gamma (1+2\alpha )}{\Gamma (1+ \alpha )^{2}}. $$

#### Proof

Identity (4.7) follows from (4.1). To show (4.8), we apply the discrete Fourier transform to obtain that

$$ \mathcal{F}\bigl(K_{+}^{\alpha }\ast K_{-}^{\alpha } \bigr) (z)=\biggl(1-\frac{1}{z}\biggr)^{\alpha }(1-z)^{\alpha }= \biggl(2-\biggl(z+\frac{1}{z}\biggr)\biggr)^{\alpha }=\mathcal{F} \bigl(K_{d}^{ \alpha }\bigr) (z) $$

for $z\in \mathbb{T}$. Since the discrete Fourier transform is one-to-one, we obtain the equality. To prove (4.9), we note that the right-hand side evaluated at $n \in \mathbb{Z}$ is equal to ${(k_{-}^{-\alpha }}*k^{\alpha })(n)$, and we have

$$ {\bigl(k_{-}^{-\alpha }}*k^{\alpha }\bigr) (n)= \sum _{j=0}^{n} k^{-\alpha }\bigl(-(n-j) \bigr)k^{- \alpha }(j) = \sum_{j=0}^{n} K^{\alpha }_{+}(n-j)K^{\alpha }_{-}(j)= \bigl(K_{+}^{ \alpha }\ast K_{-}^{\alpha }\bigr) (n), $$

and the result follows from (4.8). The spectrum is given in [37, Theorem 1.3 (iii)], and the norm of $K^{\alpha }$ is calculated in [37, Lemma 3.2]. □

An interesting consequence is the following corollary, which seems to be a new formula for binomials of noninteger entries.

#### Corollary 4.7

*Let* $\alpha \in (0,1)$ *and* $n\in \mathbb{N}_{0}$. *We have the following equality*:

$$ \binom{2\alpha }{\alpha +n}=\sum_{j=0}^{\infty } \binom{\alpha }{j+n}\binom{ \alpha }{j}. $$

#### Proof

The combinatorial equality is a straightforward consequence of the explicit expression of the kernel convolutions $K_{+}^{\alpha }$, $K_{-}^{\alpha }$, and $K^{\alpha }_{d}$. □

The following result collects the main results on the fractional discrete semigroup. For other results, see also [37].

#### Theorem 4.8

*For any* $0<\alpha <1$, *we have that the fractional discrete semigroup generated by* $-K^{\alpha }_{d}$ *is given by*

$$ e^{-zK^{\alpha }_{d}}(n) =(-1)^{n}\sum_{k=1}^{\infty }(-1)^{k} \frac{z^{k} }{k!} \frac{\Gamma (2k\alpha +1)}{\Gamma (1+k\alpha +n)\Gamma (1+k \alpha -n)}+\delta _{0}(n) $$

*for* $n\in \mathbb{Z}$ *and* $z\in \mathbb{C}$. *Moreover*:

(i) *The discrete Fourier transform of* $e^{-zK^{\alpha }_{d}}$ *is given by*
  $$ {\mathcal{F}}\bigl( e^{-zK^{\alpha }_{d}}\bigr) \bigl(e^{i\theta } \bigr)=e^{-z(4\sin ^{2}(\frac{ \theta }{2}))^{\alpha }}, \quad \theta \in [-\pi , \pi ), z \in \mathbb{C}. $$
(ii) $e^{-tK^{\alpha }_{d}}(n)\ge 0$, *and* $\Vert e^{-tK^{\alpha }_{d}}\Vert _{1}=1$ *for* $n\in \mathbb{Z}$ *and* $t\ge 0$, *that is*, *it is a Markovian semigroup*.
(iii) $\sigma (e^{-zK^{\alpha }_{d}})= \{ e^{-z(4\sin ^{2}(\frac{\theta }{2}))^{ \alpha }} : \theta \in [-\pi , \pi ) \} $.

#### Proof

The fractional discrete semigroup generated by $-K^{\alpha }_{d}$ is given in [37, Theorem 1.3]. There the entire group $(e^{-zK^{\alpha }_{d}})_{z\in \mathbb{C}}$ is written as $L_{z}^{\alpha }$. Statement (i) follows from Proposition 2.4(ii) combined with (4.7) in Theorem 4.6. The proof of (ii) is contained in [37, Theorem 1.3(v)]. Finally, to prove (iii), we use (2.9) in Theorem 2.6 and (4.7) in Theorem 4.6. □

We also apply the Lévy subordination principle (4.2) to the semigroup generated by $a=\delta _{-1}-2\delta _{0}+\delta _{1}$ to obtain the following result.

#### Corollary 4.9

*Let* $0<\alpha <1$, *and let* $f_{s,\alpha }$ *be the Lévy stable process defined by* (A.11). *For* $n\in \mathbb{Z}$ *and* $0< t<1$, *we have*

$$ \sum_{j=0}^{\infty }K^{\alpha j}_{d}(n) \frac{(-t)^{j}}{j!}= \int _{0}^{ \infty } f_{t,\alpha }(s) e^{-2s} I_{n}(2s)\,ds; $$

*in particular*, *for* $\alpha =\frac{1}{2}$,

$$ \sum_{j=0}^{\infty }K^{\frac{ j}{2}}_{d}(n) \frac{(-t)^{j}}{j!}= \int _{0}^{ \infty } \frac{t}{\sqrt{4\pi s^{3}}}e^{\frac{-t^{2}}{4s}} e^{-2s} I_{n}(2s)\,ds. $$

### The operator $(-\Delta _{dd})^{\alpha }$

Since the element $(\delta _{2}-2\delta _{0}+\delta _{-2})$ generates a uniformly bounded $C_{0}$-semigroup, we consider the fractional power $(\delta _{2}-2\delta _{0}+\delta _{-2})^{\alpha }$ for $0<\alpha <1$. For simplicity, we write $K^{\alpha }_{dd}$ instead of $(\delta _{2}-2\delta _{0}+\delta _{-2})^{\alpha }$.

#### Theorem 4.10

*Let* $0<\alpha <1$.

(i) *We have*
  $$ K^{\alpha }_{dd}(n)= \frac{\Gamma (2\alpha +1)}{\Gamma (1+\alpha +\frac{n}{2})\Gamma (1+\alpha -\frac{n}{2})} \cos \biggl( \frac{n}{2}\pi \biggr), \quad n\in \mathbb{Z}. $$
(ii) $K^{\alpha }_{dd}(2n)=K^{\alpha }_{d}(n)$ *and* $K^{\alpha }_{dd}(2n-1)=0$ *for* $n\in \mathbb{Z}$.
(iii) ${\Vert K^{\alpha }_{dd}\Vert _{1}=2 \frac{\Gamma (1+2\alpha ) }{\Gamma (1+\alpha )^{2}}}$.
(iv) ${\mathcal{F}}( K^{\alpha }_{dd})(e^{i\theta })= (4\sin ^{2}(\theta ))^{ \alpha }$ *for* $\theta \in [-\pi , \pi )$, *and* $\sigma ( K^{\alpha }_{dd})=[0,4^{\alpha }]$.

#### Proof

(i) For $n\in \mathbb{Z}$, we have that

$$\begin{aligned} K^{\alpha }_{dd}(n) =&\frac{1}{2\pi } \int _{-\pi }^{\pi }\bigl(4\sin ^{2}( \theta ) \bigr)^{\alpha }e^{-in\theta }\,d\theta = \frac{4^{\alpha }}{\pi } \int _{0}^{\pi }\sin ^{2\alpha }(\theta ) \cos (n\theta )\,d\theta \\ =& \frac{\Gamma (2\alpha +1)}{\Gamma (1+\alpha +\frac{n}{2})\Gamma (1+\alpha -\frac{n}{2})} \cos \biggl(\frac{n}{2}\pi \biggr), \end{aligned}$$

where we have applied [31, Formula 3.631 (8)]. Parts (ii), (iii), and (iv) are straightforward from part (i). □

Now we consider the entire group $(e^{-zK^{\alpha }_{dd}})_{z\in \mathbb{C}}$ generated by $-K^{\alpha }_{dd}$.

#### Theorem 4.11

*For* $0<\alpha <1$, *we have*:

(i) $e^{-zK^{\alpha }_{dd}}(n)=\cos (\frac{n}{2}\pi )\sum_{k=1}^{\infty }(-1)^{k} \frac{z^{k}}{k!} \frac{\Gamma (2k\alpha +1)}{\Gamma (1+k\alpha +\frac{n }{2})\Gamma (1+k\alpha -\frac{n}{2})}+\delta _{0}(n)$, $n\in \mathbb{Z} $.
(ii) $e^{-zK^{\alpha }_{dd}}(2n)=e^{-zK^{\alpha }_{d}}(n)$ *and* $e^{-zK^{\alpha }_{dd}}(2n-1)=0$ *for* $n\in \mathbb{Z}$.
(iii) $e^{-tK^{\alpha }_{dd}}(n)\ge 0$ *and* $\Vert e^{-tK^{\alpha }_{dd}}\Vert _{1}=1$ *for* $n\in \mathbb{Z}$ *and* $t\ge 0$, *that is*, *it is a Markovian semigroup*.
(iv) ${\mathcal{F}}( e^{-zK^{\alpha }_{dd}})(e^{i\theta })=e^{-z(4\sin ^{2}({ \theta }))^{\alpha }}$ *for* $\theta \in [-\pi , \pi )$, *and*
  $$ \sigma \bigl( e^{-zK^{\alpha }_{dd}}\bigr)=\sigma \bigl( e^{-zK^{\alpha }_{dd}}\bigr)= \bigl\{ e^{-zu^{\alpha }}\mid u\in [0,4]\bigr\} . $$

#### Proof

By [47, Theorem 1, p. 263] we have that

$$ e^{-tK^{\alpha }_{dd}}(n)= \int _{0}^{\infty }f_{t,\alpha }(s)e^{-2s}I_{\frac{n }{2}}(2s) \,ds\chi _{2\mathbb{Z}}(n)=e^{-tK^{\alpha }_{d}}\biggl(\frac{n}{2}\biggr) \chi _{2\mathbb{Z}}(n), $$

and we conclude equalities (i) and (ii). Parts (iii) and (iv) are proved from similar properties of $e^{-zK^{\alpha }_{d}}$. □

#### Remark 4.12

The spectrum of the discrete fractional Laplacian $-(-\Delta _{d})^{\alpha }$ is determined by $\sigma (-(-\Delta _{d})^{\alpha }) =[-4^{\alpha },0]$, recovering the result of Lizama and Roncal [37], Theorem 1.3(iii). A similar result is obtained for the operator $-(-\Delta _{dd})^{\alpha }$ given by $\sigma (-(-\Delta _{dd})^{\alpha }) =[-4^{\alpha },0]$. For the discrete fractional difference operators −Δ and ∇, we have $\sigma ((-\Delta )^{\alpha }) =[-(1+e^{i\mathbb{T}})^{\alpha }]$ and $\sigma (\nabla ^{\alpha }) =[-(1+e^{i\mathbb{T}})^{\alpha }]$, respectively.

## Fundamental solutions for semidiscrete evolution equations

In this section, we consider the operator $Bf(n):=(b \ast f)(n)$ with $b \in \ell ^{1}(\mathbb{Z})$, $f\in \ell ^{p}(\mathbb{Z})$, $p\in [1,\infty ]$, and $n\in \mathbb{Z}$. Our objective is obtaining a fundamental representation of solutions for the following semidiscrete fractional evolution equation:

$$ \textstyle\begin{cases} \mathbb{D}_{t}^{\beta }u(n,t)=Bu(n,t)+g(n,t), &n\in \mathbb{Z}, t>0, \\ u(n,0)=\varphi (n),\qquad u_{t}(n,0)= \phi (n),&n\in \mathbb{Z}, \end{cases} $$

where $\beta \in (0,2]$. For a sufficiently regular function *v*, we denote by $\mathbb{D}_{t}^{\beta }$ the Caputo derivative of order *β* given by

$$ \mathbb{D}_{t}^{\beta }v(t)=\frac{1}{\Gamma (1-\beta )} \int _{0}^{t}(t-s)^{- \beta }v^{\prime }(s) \,ds=\bigl(g_{1-\beta }\ast v^{\prime }\bigr) (t), \quad t>0, $$

for $0<\beta <1$ and

$$ \mathbb{D}_{t}^{\beta }v(t)=\frac{1}{\Gamma (2-\beta )} \int _{0}^{t}(t-s)^{1- \beta }v^{\prime \prime }(s) \,ds=\bigl(g_{2-\beta }\ast v^{\prime \prime }\bigr) (t), \quad t>0, $$

for $1<\beta <2$. For $\beta =1$ and $\beta =2$, we consider the usual first- and second-order derivatives. Note that

$$ \lim_{\beta \to 1^{-}}\mathbb{D}_{t}^{\beta }v(t)=v'(t), \qquad \lim_{ \beta \to 2^{-}}\mathbb{D}_{t}^{\beta }v(t)=v''(t), \quad t>0; $$

however,

5.1 $$ \lim_{\beta \to 0^{+}}\mathbb{D}_{t}^{\beta }v(t)=v(t)-v(0), \qquad \lim_{\beta \to 1^{+}}\mathbb{D}_{t}^{\beta }v(t)=v'(t)-v'(0), \quad t>0; $$

see, for example, [17, 30].

To begin with, we consider the semidiscrete Cauchy problem (1.1) given in the introduction,

5.2 $$ \textstyle\begin{cases} \partial _{t} u(n,t)=Bu(n,t)+g(n,t), &n\in \mathbb{Z}, t>0, \\ u(n,0)=\varphi (n),&n\in \mathbb{Z}, \end{cases} $$

and its fundamental solution, which is obviously given by Duhamel’s formula

$$ u(n,t)= e^{Bt}\varphi (n) + \int _{0}^{t} e^{B(t-s)}g(n,s)\,ds ,\quad n \in \mathbb{Z}, t\geq 0. $$

Analogously, in the case of the second-order semidiscrete Cauchy problem

5.3 $$ \textstyle\begin{cases} \partial _{tt} u(n,t)=Bu(n,t)+g(n,t), &n\in \mathbb{Z}, t>0, \\ u(n,0)=\varphi (n),&n\in \mathbb{Z}, \\ u(n,0)=\psi (n),&n\in \mathbb{Z}, \end{cases} $$

we have that the fundamental solution is given by D’Alembert formula

$$ u(n,t)= \operatorname{Cos}(t,B)\varphi (n) + \operatorname{Sin}(t,B) \psi (n) + \int _{0}^{t} \operatorname{Sin}(t-s,B)f(s)\,ds, $$

where $\operatorname{Cos}(t,B)$ and $\operatorname{Sin}(t,B)$ are generated by *B*.

We now consider fractional in time generalizations. Given $0<\beta \leq 1$, we first consider the equation

5.4 $$ \textstyle\begin{cases} \mathbb{D}_{t}^{\beta }u(n,t)=Bu(n,t)+g(n,t), &n\in \mathbb{Z}, t>0, \\ u(n,0)=\varphi (n), &n\in \mathbb{Z}. \end{cases} $$

We recall that $E_{\alpha , \beta }(b)$ (with $b \in \ell ^{1}(\mathbb{Z})$) is the vector-valued Mittag-Leffler function given in Definition 2.2. The main result is the following theorem.

### Theorem 5.1

*Let* $\varphi , \phi \in \ell ^{p}(\mathbb{Z})$, *and let* $g:\mathbb{Z}\times \mathbb{R}_{+} \rightarrow \mathbb{C}$ *be such that for each* $t \in \mathbb{R}_{+}$, $g(\cdot ,t) \in \ell ^{p}(\mathbb{Z})$, *and* $\sup_{s \in [0,t]}\|g(\cdot ,s)\|_{p}<\infty $ *with* $1\le p\le \infty $.

(i) *For* $0<\beta <1$, *the function*
  $$\begin{aligned} u(n,t)&=\bigl(E_{\beta , 1}\bigl(t^{\beta }b\bigr)\ast \varphi \bigr) (n) \\ &\quad{}+ \int _{0}^{t}(t-s)^{\beta -1} \bigl(E_{\beta , \beta }\bigl((t-s)^{\beta }b\bigr) \ast g(\cdot ,s) \bigr) (n)\,ds, \quad n\in \mathbb{Z}, \end{aligned}$$
  *is the unique solution of the initial value problem* (5.4). *Moreover*, $u(\cdot ,t)$ *belong to* $\ell ^{p}(\mathbb{Z})$ *for* $t>0$.
(ii) *For* $1<\beta <2$, *the function*
  $$\begin{aligned} u(n,t)&=\bigl(E_{\beta , 1}\bigl(t^{\beta }b\bigr)\ast \varphi \bigr) (n)+t\bigl(E_{ \beta , 2}\bigl(t^{\beta }b\bigr)\ast \phi \bigr) (n) \\ &\quad{}+ \int _{0}^{t}(t-s)^{\beta -1} \bigl(E_{\beta , \beta }\bigl((t-s)^{\beta }b\bigr) \ast g(\cdot ,s) \bigr) (n)\,ds, \quad n\in \mathbb{Z}, \end{aligned}$$
  *is the unique solution of the initial value problem* (1.5). *Moreover*, $u(\cdot ,t)$ *belong to* $\ell ^{p}(\mathbb{Z})$ *for* $t>0$.

### Proof

Since the algebra $\ell ^{1}(\mathbb{Z})$ is semisimple (see Theorem 2.1), the formulae in (i) and (ii) are direct consequences of the scalar identities, which in case $0<\alpha <1$ can be found in [36, Sect. 3.3, formula (8)] combined with [36, Sect. 1.2]. The case $1<\alpha <2$ follows from [36, Sect. 3.3, formula (11)]. See also the references therein. □

### Remark 5.2

Now we consider the behavior of the solution as *β* tends to the integer parameter, that is, $\beta =1,2$. For simplicity, we consider the homogeneous case $g=0$. As $\beta \to 1^{-}$, the solution of equation (1.4) converges to semigroup family operators $E_{1,1}(tb)$, and as $\beta \to 2^{-}$, the solution of equation (1.5),

$$ u(\cdot ,t)= E_{\beta , 1}\bigl(t^{\beta }b\bigr)\ast \varphi +tE_{\beta , 2}\bigl(t^{\beta }b\bigr)\ast \phi , \quad t>0, $$

converges to unique mild solution of second-order Cauchy problem, that is, the sum of a cosine function and a sine function generated by *b*; see [10, Corollary 3.14.8].

However, as in the scalar case, as $\beta \to 1^{+}$, the solution of equation (1.5) converges to

$$ u(\cdot ,t)=E_{1,1}(bt)+tE_{1,2}(tb), \quad t>0. $$

Note that this function is a solution of the following first-order modified Cauchy problem:

$$ \textstyle\begin{cases} v'(n,t)=Bv(n,t)+\phi (n), &n\in \mathbb{Z}, t>0, \\ v(n,0)=\varphi (n),&n\in \mathbb{Z}, \end{cases} $$

for $\phi , \varphi \in \ell ^{p}(\mathbb{Z})$. This fact is in accordance with the interpolation property of the Caputo fractional derivative; see (5.1).

The fundamental solutions $u_{\beta ,1}$ for systems (1.4) and (1.5) are obtained by requiring that the initial value *ψ* and the initial velocity *ϕ* be the sequences $\psi =\delta _{0}$ and $\phi =0$. In the case $1<\beta \le 2$ (including the wave equation), a second fundamental solution $u_{\beta ,2}$ is given by $\psi =0$ and $\phi =\delta _{0}$; see [26, Remark 3.2]. A consequence of Theorems 5.1 and 2.5 is the following subordination theorem for fundamental solutions, which extends [26, Corollary 3.5].

### Corollary 5.3

*Let* $u_{\beta ,1}$ *and* $u_{\beta ,2}$ *be the fundamental solutions of problems* (1.4) *and* (1.5), *and let* $\Psi _{\alpha }$ *be the Wright function defined by* (A.1).

(i) *Let* $0<\beta <1$. *Then*
  $$ u_{\beta ,1}(n,t)= \int _{0}^{\infty }\Phi _{\beta }(\tau )u_{1,1}\bigl(n, \tau t^{\beta }\bigr)\,d\tau , \quad n \in \mathbb{Z}, t>0. $$
(ii) *Let* $1<\beta <2$. *Then*
  $$\begin{aligned}& u_{\beta ,1}(n,t) = \int _{0}^{\infty } \Phi _{\frac{\beta }{2}}(\tau )u_{2,1}\bigl(n, \tau t^{\frac{\beta }{2}}\bigr)\,d\tau , \\& u_{\beta ,2}(n,t) = \int _{0}^{t}\frac{(t-u)^{\frac{-\beta }{2}}}{\Gamma (1-\frac{\beta }{2})} \int _{0}^{\infty } \Phi _{\frac{\beta }{2}}( \tau )u_{2,2}\bigl(n,\tau u^{\frac{\beta }{2}}\bigr)\,d\tau \,du \end{aligned}$$
  *for* $n \in \mathbb{Z}$ *and* $t>0$.

### Remark 5.4

The Wright function $\Phi _{\frac{1}{3}}$ can be expressed in terms of the Airy function $Ai(z)$, that is,

$$ \Phi _{\frac{1}{3}}(z)=3^{\frac{2}{3}}Ai \biggl( \frac{z}{3^{\frac{1}{3}}} \biggr), \quad z\in \mathbb{C}; $$

see, for example, [28]. A integral representation of the Airy function is given by the improper Riemann integral:

$$ Ai(x)=\frac{1}{\pi } \int _{0}^{\infty }\cos \biggl(\frac{t^{3}}{3} + x t \biggr)\,dt,\quad x \in \mathbb{R}. $$

This function appears in several applied problems, in particular, in the Schrödinger equation of quantum physics and in optics (study of caustics); see more details in [44]. By Corollary 5.3(i) we conclude that

$$ E_{\frac{1}{3},1}\bigl(t^{\frac{1}{3}}b\bigr) (n)=3^{\frac{2}{3}} \int _{0}^{\infty }Ai\biggl(\frac{ \tau }{3^{\frac{1}{3}}} \biggr)e^{\tau t^{\frac{1}{3}} b}(n)\,d\tau , \quad n \in \mathbb{Z}, t>0, $$

for $b\in \ell ^{1}(\mathbb{Z})$.

The particular case of Theorem 5.1 with $B=-(-A)^{\alpha }$, where *A* is the infinitesimal generator of an uniformly bounded $C_{0}$-semigroup in ${\mathcal{B}}(\ell ^{p}(\mathbb{Z}))$, has received a special attention. In [34, Theorem 3.3] and [26, Theorem 3.1] the time/space fractional evolution equations (1.4) and (1.5) of orders $0<\beta \leq 1$ and $1<\beta \leq 2$, respectively, are solved, where $B=-(-\Delta _{d})^{\alpha }$, and $\Delta _{d}$ is the discrete Laplacian operator. Both proofs are based on the explicit expressions of vector-valued Mittag-Leffler functions $E_{\beta , 1}(-t^{\beta }K^{\alpha }_{d})$, $E_{\beta , 2}(-t^{\beta }K^{\alpha }_{d})$, and $E_{\beta , \beta }(-t^{\beta }K^{\alpha }_{d})$. As a consequence of the results in Sect. 4, we can easily give a general version, which extends both results.

### Corollary 5.5

*Let* $\varphi , \phi \in \ell ^{p}(\mathbb{Z})$, *and let* $g:\mathbb{Z}\times \mathbb{R}_{+} \rightarrow \mathbb{C}$ *be such that for each* $t \in \mathbb{R}_{+}$, $g(\cdot ,t) \in \ell ^{p}(\mathbb{Z})$ *and* $\sup_{s \in [0,t]}\|g(\cdot ,s)\|_{p}<\infty $ *with* $1\le p\le \infty $. *For* $a\in \ell ^{1}(\mathbb{Z})$ *generating a uniformly continuous semigroup in* $\ell ^{1}(\mathbb{Z})$, *we write* $(-a)^{\alpha }$ *for the fractional powers given in Definition *4.1*and* $B(f):=-(-a)^{\alpha }\ast f$ *for* $f\in \ell ^{p}(\mathbb{Z})$ *and* $0<\alpha <1 $. *Then the same representation of the fundamental solutions given in Theorem *5.1*with* $b=-(-a)^{\alpha }$ *holds*.

## Applications

We study some concrete examples that appear in various applied fields.

### The discrete Nagumo equation

Let us consider the linear part of the discrete Nagumo equation, which can be written as follows:

6.1 $$ \textstyle\begin{cases} \partial _{t} u(n,t)= \Delta _{d} u(n,t) -ku(n,t), &n\in \mathbb{Z}, t>0, \\ u(n,0)=\varphi (n),&n\in \mathbb{Z}, \end{cases} $$

where $0< k<1/2$. The discrete Nagumo equation is used as a model for the spread of genetic traits and for the propagation of nerve pulses in a nerve axon, neglecting recovery; see [48] and references therein. Using Theorem 3.4(3), we obtain

$$ \sigma \bigl(e^{t(\Delta _{d}-kI)}\bigr)= e^{t \sigma (\Delta _{d}-kI) } = \bigl\{ e^{ts} : t\geq 0, -4-k\leq s \leq -k \bigr\} . $$

This implies that the unique solution of equation (6.1) is uniformly asymptotically stable, that is,

$$ u(n,t)= e^{t(\Delta _{d}-kI)}\varphi (n) \to 0\quad \text{as } t\to \infty . $$

Moreover, using Theorem 3.4(4) and the semigroup property, we can obtain the following representation of the fundamental solution:

$$\begin{aligned} u(n,t) &= e^{-tkI}e^{t\Delta _{d}}\varphi (n) := \bigl(e^{-tkI}*e^{t \Delta _{d}}* \varphi \bigr) (n) = \sum_{j=0}^{n} \bigl(e^{-tkI}*e^{t\Delta _{d}}\bigr) (n-j) \varphi (j) \\ & = e^{-2t} \sum_{j=0}^{n} \sum _{l=0}^{n-j}\frac{(-kt)^{l}}{l!} I_{n-j-l}(2t) \varphi (j). \end{aligned}$$

Since $\sigma ( -(-\Delta _{d})^{\alpha })=[-4^{\alpha },0]$ (see Remark 4.12), we have that the same asymptotic behavior also holds for the fundamental solution of the fractional Laplacian version for the discrete Nagumo equation [37, Sect. 7]:

$$ \textstyle\begin{cases} \partial _{t} u(n,t)= -(-\Delta _{d})^{\alpha } u(n,t) -ku(n,t), &n \in \mathbb{Z}, t>0, \\ u(n,0)=\varphi (n),&n\in \mathbb{Z}. \end{cases} $$

### The semidiscrete transport equation associated with the *r*-difference operator

Let us consider the semidiscrete transport equation

6.2 $$ \textstyle\begin{cases} \partial _{t} u(n,t)= {_{r}\Delta } u(n,t), &n\in \mathbb{Z}, t>0, \\ u(n,0)=\varphi (n),&n\in \mathbb{Z}, \end{cases} $$

where $r>0$, and _*r*_Δ is the *r*-forward difference operator defined by ${_{r}\Delta }f(n):= f(n+1)-rf(n)$; see [7, Sect. 5.5]. Observe that ${_{r}\Delta }= \Delta + (1-r)I $, where *I* is the identity operator. Then by perturbation semigroup theory the unique solution of (6.2) has the form $u(n,t)=e^{t(\Delta +(1-r)I)}\varphi (n)$, $n \in \mathbb{Z}$. By the spectral mapping (2.9) in Theorem 2.6 we obtain that

$$ \sigma \bigl(e^{t(\Delta +(1-r)I)}\bigr)= e^{t\sigma (\Delta + (1-r)I))}. $$

Hence by Theorem 3.2(3) we deduce that $\sigma (\Delta +(1-r)I)=\{ z\in \mathbb{T} : |z+r|=1 \} $. Therefore for any $r>1$, we have that the upper bound of the spectrum of $B=\Delta + (1-r)I $ is negative, that is,

$$ \omega _{\sigma }\bigl(\Delta + (1-r)I\bigr):= \sup \bigl\{ \Re z : z \in \sigma \bigl( \Delta +(1-r)I\bigr) \bigr\} < 0, $$

and, consequently, we obtain that for any $r>1$, the unique solution of equation (6.2) is uniformly asymptotically stable, that is,

$$ \bigl\Vert e^{{_{r}\Delta } t}\varphi \bigr\Vert \to 0 \quad \text{as } t \to \infty , $$

uniformly with respect to $\|\varphi \|\leq 1$. Of course, this result can be also directly deduced from Theorem 3.2(5). Analogously, using the fact that

$$ \Re \bigl(r-e^{it}\bigr) >0 \quad \text{implies}\quad \Re \bigl( \bigl(r-e^{it}\bigr)^{\alpha }\bigr)>0 $$

for any $0<\alpha <1$ and $r>1$, we can deduce from Theorem 4.4 that the same property of asymptotic stability remains true for the unique solution of the fractional semidiscrete transport equation

6.3 $$ \textstyle\begin{cases} \partial _{t} u(n,t)=-(-{_{r}\Delta })^{\alpha } u(n,t), &n\in \mathbb{Z}, t>0, \\ u(n,0)=\varphi (n),&n\in \mathbb{Z}. \end{cases} $$

### The De Juhasz equation

We consider the following semidiscrete equation:

6.4 $$ \textstyle\begin{cases} \partial _{tt} u(n,t)= \Delta _{d} u(n,t) - 2ku(n,t), &n\in \mathbb{Z}, t>0, \\ u(n,0)=\varphi (n),&n\in \mathbb{Z}, \\ u_{t}(n,0)=\psi (n),&n\in \mathbb{Z}, \end{cases} $$

where $k>0$. This equation can be found in the seminal paper of Bateman [16] in connection with surges in springs and connected systems of springs. We call it the De Juhasz equation because, according Bateman’s paper, De Juhasz deduced for the first time the modeling of such equation in mechanical theory. Following Bateman’s paper, this semidiscrete equation is obtained when the concentrated masses on a light string are mounted on springs arranged either along a straight line or on the circumference of a circle or helix [16, Sect. 5, formula (5.1)]. Applying Theorem 3.4 and considering the operator $B=\Delta _{d} -2kI$, we obtain

$$ \sigma (\Delta _{d} -2kI) = [-4-2k, -2k], $$

and therefore

$$ \sigma \bigl(\operatorname{Cos}(t,B)\bigr)= \bigl\{ \cos (t\sqrt{s}) : s \in [2k,4+2k] \bigr\} . $$

In particular, this implies that on the Hilbert space $\ell ^{2}(\mathbb{Z})$, we have $\|\operatorname{Cos}(t)\|\leq 1$, and, consequently, the unique solution of (6.4) when $\psi \equiv 0$ must be bounded. This extends the previous result of Bateman [16, Sect. 5], who studied (6.4) with the initial conditions $\psi \equiv 0$ and $\varphi (n)=\delta _{0}(n)$.

### The Caputo–Fabrizio derivative

We recall that given a sufficiently regular function *u* and $0<\alpha <1$, the Caputo–Fabrizio derivative of order *α* is defined as [19]

$$ {}^{CF}D^{\alpha }u(t)= \frac{1}{1-\alpha } \int _{0}^{t} e^{ \frac{-\alpha }{1-\alpha }(t-s)}u'(s) \,ds. $$

Note that the Caputo–Fabrizio derivative has been very recently used to propose a new mathematical modeling of human liver [12], HIV [13], parallel RCL circuits [8], the Rubella disease model [14], epidemic childhood diseases [11], and COVID-19 [15]. However, with the exception of the implicit solution for the linear model in the scalar case, proposed by Losada and Nieto in [38], so far no explicit formulas have been proposed for the solution of the fractional Cauchy problem in the context of Banach algebras.

We consider the equation

6.5 $$ \textstyle\begin{cases} {}^{CF}D^{\alpha }_{t} u(n,t)=Bu(n,t)+g(n,t), &n\in \mathbb{Z}, t \geq 0, \\ u(n,0)=\varphi (n),&n\in \mathbb{Z}, \end{cases} $$

where we recall that $Bf(n):=(b \ast f)(n)$ with $b \in \ell ^{1}(\mathbb{Z})$, $f\in \ell ^{p}(\mathbb{Z})$, $p\in [1,\infty ]$, and $n\in \mathbb{Z}$. Since *B* is bounded, assuming that $\frac{1}{1-\alpha } \in \rho (B)$, we obtain the following representation for the solution of (6.5):

6.6 $$ u(n,t)=T(t)\varphi (n) + \int _{0}^{t} T(t-s)h(n,s)\,ds, \quad n \in \mathbb{Z}, t>0, $$

where

6.7 $$ T(t):=e^{\frac{\alpha }{1-\alpha }(1-(1-\alpha ) B)^{-1}t}e^{- \frac{\alpha }{1-\alpha }t}, \quad t\geq 0, $$

and

6.8 $$ h(n,t):=\bigl(I-(1-\alpha ) B\bigr)^{-1}\bigl[(1-\alpha )g_{t}(n,t) + \alpha g(n,t)\bigr]. $$

Indeed, since *B* is bounded, from [38, Proposition 2] and taking into account [38, formula (8) and the explicit formula for $M(\alpha )$ given in Remark p. 89] we know that the unique solution of problem (6.5) is given by the unique solution of the problem

$$\begin{aligned} u_{t}(n,t) &= \alpha B\bigl(I-(1-\alpha ) B\bigr)^{-1}u(n,t) \\ &\quad{}+ \bigl(I-(1-\alpha ) B\bigr)^{-1}\bigl[(1-\alpha )g_{t}(n,t) + \alpha g(n,t)\bigr] \end{aligned}$$

(note that there is a small but important misprint in [38, p. 90, l. 16], where we must read $\tilde{\sigma }'(t)$ instead of $\tilde{\sigma }(t)$). Using the identity

$$ (1-\alpha ) B\bigl(1-(1-\alpha ) B\bigr)^{-1}= \bigl(I-(1-\alpha ) B \bigr)^{-1} -I, $$

we obtain

$$\begin{aligned} u_{t}(n,t) &= \biggl[\frac{\alpha }{1-\alpha }\bigl(I-(1-\alpha ) B \bigr)^{-1} - \frac{\alpha }{1-\alpha }\biggr]u(n,t) \\ & \quad + \bigl(I-(1-\alpha ) B\bigr)^{-1}\bigl[(1-\alpha )g_{t}(n,t) + \alpha g(n,t)\bigr], \end{aligned}$$

and hence (6.6) follows by using the classical Duhamel formula.

In terms of Banach algebras, this result reads as follows. The proof is similar to that of Theorem 5.1 and is therefore omitted.

#### Theorem 6.1

*Let* $\varphi \in \ell ^{p}(\mathbb{Z})$, *and let* $g:\mathbb{Z}\times \mathbb{R}_{+} \rightarrow \mathbb{C}$ *be such that for each* $t \in \mathbb{R}_{+}$, $g(\cdot ,t) \in \ell ^{p}(\mathbb{Z})$, $t\to g(t,n)$ *is differentiable and* $\sup_{s \in [0,t]}\|g(\cdot ,s)\|_{p}<\infty $ *with* $1\le p\le \infty $. *For* $0<\alpha <1$, *assume that* $(1-\alpha ) \|b\|_{1} <1$. *Then the function*

$$\begin{aligned} u(n,t)&=\bigl(e^{\alpha b*(\delta _{0}-(1-\alpha ) b)^{-1}t} \ast \varphi \bigr) (n) \\ &\quad{}+ \int _{0}^{t} \bigl(e^{\alpha b*(\delta _{0}-(1-\alpha ) b)^{-1}(t-s)} \ast h( \cdot ,s) \bigr) (n)\,ds, \quad n\in \mathbb{Z}, \end{aligned}$$

*is the unique solution of the initial value problem* (6.5), *where*

$$ h(n,t)= \bigl(\delta _{0}-(1-\alpha ) b\bigr)^{-1}* \bigl[(1-\alpha )g_{t}(n,t) + \alpha g(n,t)\bigr]. $$

*Moreover*, $u(\cdot ,t)$ *belong to* $\ell ^{p}(\mathbb{Z})$ *for* $t>0$.

#### Remark 6.2

Note that Theorems 3.2 and 3.3 say that for the discrete operators −Δ and ∇, the condition $(1-\alpha ) \|b\|_{1} <1$ implies $1/2<\alpha <1$, whereas Theorems 3.4 and 3.5 tell us that for the operators $\Delta _{d}$ and $\Delta _{dd}$, we must have $3/4<\alpha <1$.

## Applications to special functions

In this section, we present some new formulae obtained as applications of the results proved in this paper. We give expressions of generalized Mittag-Leffler functions for concrete fractional powers. We also interpret some known formulas in terms of the Weierstrass subordination formula. Finally, the application of subordination principle to Wright function of some concrete difference operators allows us to obtain some new integral formulae for Bessel and Wright functions.

### Generalized Mittag-Leffler functions for fractional powers

As we have seen in Theorem 5.1, combinations of vector-valued Mittag-Leffler functions in $\ell ^{1}(\mathbb{Z})$ give the solutions of fractional evolution equations (1.4) and (1.5) with $b\in \ell ^{1}(\mathbb{Z})$. In the case of fractional powers of elements in $\ell ^{1}(\mathbb{Z})$, that is, $b=-(-a)^{\alpha }$, we present Corollary 5.5. Taking into account Sects. 4.1, 4.2, and 4.3, we can give some particular representations of the associated Mittag-Leffers functions.

#### Theorem 7.1

*The generalized Mittag*-*Leffler functions* $E_{\gamma ,\beta }$ *for* $K^{\alpha }_{+}$, $K^{\alpha }_{-}$, $K^{\alpha }_{d}$, *and* $K^{\alpha }_{dd}$ *with* $\gamma ,\beta >0$ *and* $0<\alpha <1$ *are given as follows*:

(i) $E_{\gamma ,\beta }(-t^{\gamma }K^{\alpha }_{+})(n) = \sum_{j=0}^{ \infty }(-1)^{j}\frac{t^{\gamma j} }{\Gamma (\gamma j+\beta )}k^{- \alpha j}(-n)\chi _{-\mathbb{N}_{0}}(n) $;
(ii) $E_{\gamma ,\beta }(-t^{\gamma }K^{\alpha }_{-})(n) = \sum_{j=0}^{ \infty }(-1)^{j}\frac{t^{\gamma j} }{\Gamma (\gamma j+\beta )}k^{- \alpha j}(n)\chi _{\mathbb{N}_{0}}(n)$;
(iii) $E_{\gamma ,\beta }(-t^{\gamma }K^{\alpha }_{d})(n) = (-1)^{n} \sum_{j=0}^{\infty }\frac{(-1)^{j}t^{\gamma j}}{\Gamma (\gamma j+ \beta )} \frac{\Gamma (2\alpha j+1)}{\Gamma (\alpha j+n+1)\Gamma (\alpha j-n+1)}$;
(iv) $E_{\gamma ,\beta }(-t^{\gamma }K^{\alpha }_{dd})(n)= \cos (\frac{n }{2}\pi )\sum_{j=0}^{\infty }\frac{(-1)^{j}t^{\gamma j}}{\Gamma ( \gamma j+\beta )} \frac{\Gamma (2\alpha j+1)}{\Gamma (\alpha j+\frac{n}{2}+1)\Gamma (\alpha j-\frac{n}{2}+1)}$.

### Weierstrass formula

A relation between cosine functions and semigroups generated by an element $a\in \ell ^{1}(\mathbb{Z})$ is established by the Weierstrass formula in (2.6). Now we apply this formula to concrete finite difference operators.

1. For $a= \delta _{-1}-\delta _{0}$ or $a= \delta _{1}-\delta _{0}$, we obtain that
  $$ \frac{1}{\sqrt{ t}} \int _{0}^{\infty }e^{-\frac{s^{2}}{4t}} \biggl( \frac{s}{2} \biggr)^{n+\frac{1}{2}}J_{n-\frac{1}{2}}(s)\,ds=t^{n}e^{-t}, \quad n\in \mathbb{N}_{0}, t>0. $$
  This formula is a particular case of the general formula
  $$ \int _{0}^{\infty }e^{-p^{2}t^{2}} t^{\nu +1}J_{\nu }(at) \,dt= \frac{a^{\nu }}{(2p^{2})^{\nu +1}}e^{-\frac{a^{2}}{4p^{2}}}, \quad p\in \mathbb{R}, a>0, $$
  for $\Re (\nu )>-1$, [45, Sect. 13.3, formula (4)].
2. For $a=\delta _{-1}-2\delta _{0}+\delta _{1}$ or $a=\delta _{-2}-2\delta _{0}+\delta _{2}$, we have that
  $$ \frac{1}{\sqrt{\pi t}} \int _{0}^{\infty }e^{-\frac{s^{2}}{4t}}J_{2n}(2s) \,ds=e^{-2t}I_{n}(2t), \quad n\in \mathbb{Z}, t\in \mathbb{R}. $$
  As it is commented in [37, Remark 3], this formula is a particular case of the general equality
  $$ \int _{0}^{\infty }e^{-pt^{2}} J_{\nu }(at) \,dt= \frac{1}{2}\sqrt{\frac{\pi }{p}}e^{-\frac{a^{2} }{8p}}I_{\frac{\nu }{2}} \biggl(\frac{a^{2}}{8p}\biggr), \quad p>0, a>0, $$
  for $\Re (\nu )>-1$ [45, Sect. 13.3, formula (5)].

### Subordination principle on Wright function

In this subsection, we apply Corollary 5.3 to finite difference operators. We obtain some known formulae, but others seem to be new; see (7.1), (7.2), and (7.3). They give some interesting new connections between the Wright and Bessel functions.

Take $a= \delta _{-1}-\delta _{0}$ or $a= \delta _{1}-\delta _{0}$ in Corollary 5.3.

(i) For $0<\beta <1$, $t\in \mathbb{C}$, and $n\in \mathbb{N}_{0}$, we have
  $$ E_{\beta ,1}^{(n)}(t)=\sum_{j=0}^{\infty } \frac{(j+n)!}{j!} \frac{t^{ j}}{\Gamma (\beta (j+n) +1)} = \int _{0}^{\infty } \Phi _{ \beta }(\tau ) e^{\tau t} \tau ^{n}\,d\tau . $$
  For $n=0$, we obtain formula (A.4) and for $t=0$, formula (A.2). For $\beta =\frac{1}{3}$, we obtain the following integral formula for the Airy function:
  $$ E_{\frac{1}{3},1}^{(n)}(t)=\sum_{j=0}^{\infty } \frac{(j+n)!}{j!}\frac{t^{j} }{\Gamma (\frac{j+n}{3} +1)} = \int _{0}^{\infty } 3^{\frac{2}{3}}Ai \biggl( \frac{\tau }{3^{\frac{1}{3}}} \biggr)e^{\tau t} \tau ^{n} \,d \tau $$
  for $n\in \mathbb{N}_{0}$ and $t\in \mathbb{C}$. For $t=0$, we get [44, formula (3.83)].
(ii) For $1<\beta <2$, $t\in \mathbb{C}$, and $n\in \mathbb{N}_{0}$, we have
  7.1 $$ (2t)^{n-\frac{1}{2}}\sum_{j=0}^{\infty } \frac{(-1)^{j} (j+n)!}{j!} \frac{t^{2 j}}{\Gamma (\beta (j+n) +1)} =\frac{\sqrt{\pi }}{2} \int _{0}^{ \infty } \Phi _{\frac{\beta }{2}}(\tau ) \tau ^{n+\frac{1}{2}} J_{n- \frac{1}{2}}(\tau t) \,d\tau . $$

In the cases $n=0$ and $n=1$, we obtain formulae (A.5).

Now take $a=\delta _{-1}-2\delta _{0}+\delta _{1}$ or $a=\delta _{-2}-2\delta _{0}+\delta _{2}$.

(i) For $0<\beta <1$, $t\in \mathbb{C}$, and $n\in \mathbb{N}_{0}$, we have
  7.2 $$ \sum_{j=0}^{\infty }(-1)^{j} \binom{2(j+n)}{j} \frac{t^{ j+n}}{\Gamma (\beta (j+n) +1)}= \int _{0}^{\infty } \Phi _{ \beta }(\tau ) e^{-2\tau t} I_{n}(2\tau t)\,d\tau . $$
  In particular, when $\beta =\frac{1}{3}$, we get the integral formula for the Airy function:
  $$ \sum_{j=0}^{\infty } (-1)^{j} \binom{2(j+n)}{j} \frac{t^{j+n}}{\Gamma (\frac{j+n}{3} +1)}= \int _{0}^{\infty }3^{ \frac{2}{3}}Ai \biggl( \frac{\tau }{3^{\frac{1}{3}}} \biggr)e^{-2\tau t}I_{n}(2 \tau t)\,d\tau $$
  for $t\in \mathbb{C}$ and $n\in \mathbb{N}_{0}$.
(ii) For $1<\beta <2$, $t\in \mathbb{C}$, and $n\in \mathbb{N}_{0}$, we have
  7.3 $$ \sum_{j=0}^{\infty }(-1)^{j} \binom{2(j+n)}{j} \frac{t^{2 (j+n)}}{\Gamma (\beta (j+n) +1)}= \int _{0}^{\infty } \Phi _{ \frac{\beta }{2}}(\tau ) J_{2n}(2\tau t)\,d\tau . $$

## Conclusions

In this paper, we investigated in an unified way fundamental solutions of the semidiscrete Cauchy problems (1.4)–(1.5) using the theory of Banach algebras, which, as we show in this paper, have considerable advantages compared to direct approaches since spectral properties and explicit representations can be easily available. We analyzed the backward and forward difference operators and the one-dimensional discrete Laplacian, a new operator, which originates in connection with crystal lattices. We have explicitly described their associated groups and cosine functions of operators. We have presented important applications to the discrete Nagumo, transport, and De Juhasz equations, which we present in this paper for the first time. We have obtained an explicit representation of the solution in Banach algebras for the fractional Cauchy problem with the new Caputo–Fabrizio fractional derivative. We have determined fundamental solutions for (1.4)–(1.5) in the setting of Banach algebras and as convolution operators. As a byproduct, we have obtained new Weierstrass formulas and a subordination principle.

## Data Availability

Not applicable.

## Appendix: Useful properties of some special functions

In this section, we present some special functions and give some known results needed in the paper.

### A.1 Wright function $\Phi _{\gamma }$

The entire Wright function is given by

A.1 $$ \Phi _{\alpha }(z):= \sum_{n=0}^{\infty } \frac{(-z)^{n}}{n! \Gamma (-\alpha n +1 -\alpha )} = \frac{1}{2\pi i} \int _{\gamma } \mu ^{\alpha -1} e^{\mu - z \mu ^{\alpha }} \,d\mu , \quad 0< \alpha < 1, $$

where *γ* is a contour that starts and ends at −∞ and encircles the origin once counterclockwise; see, for example, [28, formula (28)]. These functions were initially studied by Wright [46] in connection with the asymptotic theory of partitions. For $0<\alpha <1$, we have the following properties:

(i) $\Phi _{\alpha }(t) \geq 0$ for $t>0$.
(ii) $\int _{0}^{\infty }\Phi _{\alpha }(t)\,dt =1$.

It follows that $\Phi _{\alpha }$ is a probability density function on $\mathbb{R}_{+}$. In fact, the Wright function has been used for models in stochastic processes; see, for example, [28, 29]. In a general sense, as $\alpha \to 1^{-}$, we may interpret that $\Phi _{\alpha }\to \delta _{1}$, where $\delta _{1}$ is the Delta measure concentrated in $t=1$ ([28]).

The following formula is known for the moments of the Wright function:

A.2 $$ \int _{0}^{\infty }x^{p} \Phi _{\alpha }(x) \,dx= \frac{\Gamma (p+1)}{\Gamma (\alpha p+1)}, \quad 0< \alpha < 1, p>-1; $$

see [28].

The Mittag-Leffler function is a complex function depending on two complex parameters *α* and *β*. When the real part of *α* is strictly positive, it may be defined by the series

A.3 $$ E_{\alpha , \beta }(z) := \sum_{j=0}^{\infty } \frac{z^{ j}}{\Gamma (\alpha j + \beta )}, $$

where Γ is the gamma function. When ${\beta =1}$, it is abbreviated as ${E_{\alpha }(z)=E_{\alpha ,1}(z)}$.

An interesting fact is the following relationship between the Wright function and the Mittag-Leffler functions:

A.4 $$ E_{\alpha ,1}(z)= \int _{0}^{\infty }e^{zt}\Phi _{\alpha }(t)\,dt,\quad 0< \alpha < 1, z\in \mathbb{C}. $$

For $z\in \mathbb{C}$ and $0<\alpha <1$, we also have

A.5 $$\begin{aligned}& E_{2\alpha ,1}\bigl(-z^{2}\bigr) = \int _{0}^{\infty }\cos (zt)\Phi _{\alpha }(t)\,dt, \\& zE_{2\alpha ,1+\alpha }\bigl(-z^{2}\bigr) = \int _{0}^{\infty }\sin (zt)\Phi _{ \alpha }(t)\,dt; \end{aligned}$$

see [30, formula 2.29]. For more detail on the Wright functions, we refer to [28–30, 39, 40, 46] and the references therein.

### A.2 Bessel functions $J_{\nu }$ and $I_{\nu }$

For $\nu \in \mathbb{R}$, let $J_{\nu }$ denote the *Bessel function* defined by

A.6 $$ J_{\nu }(x)=\sum_{n=0}^{\infty } \frac{(-1)^{n}}{\Gamma (n+\nu +1)n!} \biggl(\frac{x}{2} \biggr)^{2n+\nu },\quad x\ge 0. $$

The *modified Bessel functions of the first kind* are defined by

A.7 $$ I_{\nu }(x)=\sum_{n=0}^{\infty } \frac{1}{\Gamma (n+\nu +1)n!} \biggl( \frac{x}{2} \biggr)^{2n+\nu }. $$

Furthermore, from the definition of $J_{\nu }(x)$ and $I_{\nu }(x)$ it is clear that $|J_{\nu }(x)|\leq I_{\nu }(x)$, $\nu \in \mathbb{R}_{+}$.

Directly from definition (A.7) we have that $I_{n}(x) \geq 0$, $n\in \mathbb{Z}$, $x\geq 0$. We recall some properties of the Bessel functions, the generating function, symmetry, and the Gegenbauer addition formula (see [31, formula 8.511], [9]):

1. $\sum_{n\in \mathbb{Z}} J_{n}(x)z^{n} = e^{ \frac{x}{2}(z-\frac{1}{z})}$, $z \in \mathbb{C}\setminus \{0\}$, $x\in \mathbb{C}$.
2. $J_{n}(-x)=J_{-n}(x)=(-1)^{n}J_{n}(x)$.
3. $J_{n}(x+y)=\sum_{k\in \mathbb{Z}}J_{n-k}(x)J_{k}(y)$.
4. $\sum_{n\in \mathbb{Z}} I_{n}(x)z^{n} = e^{ \frac{x}{2}(z+\frac{1}{z})}$, $z \in \mathbb{C}\setminus \{0\}$, $x\in \mathbb{C}$.
5. $I_{-n}(x)=I_{n}(x)=(-1)^{n}I_{n}(-x)$.
6. $I_{n}(x+y)=\sum_{k\in \mathbb{Z}}I_{n-k}(x)I_{k}(y)$.

Note that

$$ J_{\frac{1}{2}}(z)=\sqrt{\frac{2}{\pi z}}\sin (z), \qquad J_{\frac{-1}{2}}(z)= \sqrt{\frac{2}{\pi z}}\cos (z) $$

[31, formula 8.464].

For $\nu >-1$ and $\beta , \alpha >0$, we have

A.8 $$\begin{aligned}& \int _{0}^{\infty }e^{-\alpha t}I_{\nu }( \beta t) \,dt=\frac{\beta ^{-\nu } ( \alpha - \sqrt{\alpha ^{2}-\beta ^{2}} )^{\nu }}{\sqrt{ \alpha ^{2}-\beta ^{2}}}, \end{aligned}$$

A.9 $$\begin{aligned}& \int _{0}^{\infty }e^{-\alpha t}J_{\nu }( \beta t) \,dt=\frac{\beta ^{-\nu } ( \sqrt{\alpha ^{2}+\beta ^{2}}-\alpha )^{\nu }}{\sqrt{ \alpha ^{2}+\beta ^{2}}}; \end{aligned}$$

see, for example, [31, formula 6.611].

For $\nu >-1$ and $\beta , \alpha >0$,

A.10 $$ \int _{0}^{\infty }e^{-\alpha t}J_{\nu }( \beta t) t^{\nu +1} \,dt= \frac{(2 \alpha )(2\beta )^{\nu }\Gamma (\nu +\frac{3}{2})}{\sqrt{\pi }( \alpha ^{2}+\beta ^{2})^{\nu + \frac{3}{2}}}; $$

see, for example, [31, formula 6.623(2)]. For more information on extended forms of the Bessel function, among others, and their connections with elementary functions, see [6] and [20].

### A.3 Stable Lévy distribution $f_{t,\alpha }$

The so-called stable Lévy distribution is defined for $0<\alpha <1$ by

A.11 $$ f_{t,\alpha }(s) := \frac{1}{2\pi i} \int _{\sigma - i \infty }^{ \sigma + i \infty } e^{ z s - t z^{\alpha }} \,dz, \quad \sigma >0, t>0, s \geq 0, $$

where the branch of $z^{\alpha }$ is taken so that ${\Re }(z^{\alpha })>0$ for ${\Re }(z)>0$. We remark that there is no analytical representation for the Lévy distribution, except in the case $\alpha =\frac{1}{2}$. An explicit representation is given by

$$ f_{t,\frac{1}{2}}(s)=\frac{t}{\sqrt{4\pi s^{3}}}e^{\frac{-t^{2}}{4s}}, \quad t,s>0. $$

These functions were introduced by Bochner [18] in the study of certain stochastic processes. Yosida [47] used them systematically in the study of semigroups generated by fractional powers of uniformly bounded $C_{0}$-semigroups of linear operators. The Lévy functions are the density functions associated with the stable Lévy processes in the rotational invariant case and are related to the fractional Brownian motion. The following properties hold:

(i) For $t>0$ and $a>0$,
  A.12 $$ \int _{0}^{\infty } e^{-\lambda a} f_{t,\alpha }( \lambda ) \,d\lambda = e^{-ta^{\alpha }}.$$
(ii) $f_{t,\alpha }(\lambda ) \geq 0$, $\lambda >0$, $t>0$.
(iii) $\int _{0}^{\infty } f_{t,\alpha }(\lambda ) \,d\lambda = 1$, $t>0$.
(iv) $f_{t+s,\alpha } (\lambda )= \int _{0}^{\lambda } f_{t, \alpha }(\lambda -\mu )f_{s,\alpha }(\mu )\,d\mu $, $\lambda >0$, $t,s>0$.
(v) $\int _{0}^{\infty } e^{\lambda z} f_{\lambda ,\alpha }(t)\,d \lambda = t^{\alpha -1}E_{\alpha ,\alpha }(zt^{\alpha })$, $z \in \mathbb{C}$, $t >0$.

See, for example, [47, p. 260–262]. For the interesting property (v), we refer to [2, Theorem 3.2(iii)].
